# Morphological and trophic coupling distinguishes coral clades along a light gradient

**DOI:** 10.1002/lno.70469

**Published:** 2026-07-31

**Authors:** Taylor R. Lindsay, Hana-lei Evans, Morgan Schwartz, Kelton W. McMahon, Carlos Prada

**Affiliations:** 1 Center for Biotechnology and Life Sciences, University of Rhode Island, Kingston, Rhode Island, USA;; 2 College of Science and Engineering, James Cook University, Douglas, Queensland, Australia;; 3 Atlantic Coastal Environmental Sciences Division, U.S. EPA, Narragansett, Rhode Island, USA;; 4 Coastal Resources Center, Graduate School of Oceanography, University of Rhode Island, Narragansett, Rhode Island, USA

## Abstract

Light availability is critical to the growth and survival of most scleractinian corals due to their endosymbiotic relationship with photosynthetic dinoflagellates. Natural light gradients are expected to be associated with shifts in coral physiology, morphology, and trophic strategy. However, few studies have assessed the interplay of these biological metrics simultaneously in multiple species. We collected three coral species from each of the two scleractinian clades along a light gradient in La Parguera, Puerto Rico, and quantified corallite morphology, symbiont physiology, tissue condition, and trophic strategy. All six species experienced significant morphological change and minimal physiological change along the light gradient. Stable isotope niche analyses indicated significant decoupling between coral host and symbiont isotopic niches, consistent with a high reliance on heterotrophy in most species. This pattern was not strongly modulated by light availability in any species. However, trophic strategy differed between clades and covaried with corallite size, offering novel insights into characteristics that may differentiate clades. Our results suggest that corals change their morphologies in response to light availability to maintain physiological and trophic performance.

Light is a major structuring force for photosynthetically symbiotic scleractinian corals. At the reef scale, light availability across depth gradients influences a host of organismal responses, from shifts in the energetic relationship of coral host and symbiont to the physical morphology of the coral skeleton itself (Porter 1976; Einbinder et al. 2009; Nir et al. 2011). Corals in high light environments are disproportionately at risk of climate change-induced stressors with higher temperature and photostress closer to the sea surface. Coral communities have adapted a myriad of ways to deal with variable conditions within their environment. Some species have developed niche specialization, narrowing the range of environmental conditions to which they are specialized (Bongaerts et al. 2013; Studivan et al. 2019; Prada et al. 2022). Other species maintain phenotypic plasticity to acclimatize to their local environmental conditions upon recruitment (Todd 2008; Gomez-Campo et al. 2023; Tavakoli-Kolour et al. 2023). Regardless of their ecological strategy, corals leverage a variety of biological mechanisms, including their trophic ecology, corallite morphology, and physiology, to survive in heterogeneous and changing environments (Prada et al. 2022).

Most tropical corals are mixotrophic, meaning they acquire energy via autotrophy and heterotrophy (Furla et al. 2005; Houlbrèque and Ferrier-Pagès 2009). A symbiotic relationship with single-celled dinoflagellates in the family Symbiodiniaceae (hereafter called symbionts) living within coral tissues provides the corals with photosynthetically derived nutrients and metabolites (Muscatine et al. 1981; Furla et al. 2005). Corals can also acquire energy via heterotrophic feeding on plankton and particulate organic matter (POM) in the water column (Houlbrèque and Ferrier-Pagès 2009). Many tropical corals live in oligotrophic waters in which plankton, POM, and dissolved nutrients are often limited. Thus, symbiotic photosynthesis is commonly thought to be a dominant source of energy for scleractinian corals (Falkowski et al. 1984; Wall et al. 2020). Because photosynthesis is intrinsically tied to the survival and growth of corals, light is a critical resource on the reef. Light availability drives patterns of primary production; therefore, structuring the community composition of reefs (Porter 1976; Bongaerts et al. 2013; López-Londoño et al. 2022). To survive in competitive and stressful environments, corals can change their physiology and morphology at several spatial scales that influence light capture, transform it into energy resources, and maximize growth, survival, and fitness (López-Londoño et al. 2022; Gomez-Campo et al. 2023).

One well-documented mechanism corals use to modulate their light environment is changing colony morphology. In shallow, high light environments, scleractinian corals are generally taller, rounder, and/or have thicker branches, while corals in deep environments are characterized by flatter, broader colonies or thinner branches (Einbinder et al. 2009; Nir et al. 2011). These morphologies help to mitigate photoinhibition from excess light in shallow environments while maximizing light capture in low light environments. Similarly, there is often a shift in the corallite morphology along a light gradient. Many species have larger calices in low light environments, while in others the pattern is reversed or non-existent (Einbinder et al. 2009; Nir et al. 2011; Studivan et al. 2019). At the corallite level, a variety of morphological metrics shift along light and depth gradients. For example, in *Pocillopora verrucosa*, 60% of microstructures and 25% of macrostructures of coral skeletons changed across a depth gradient (Soto et al. 2018). However, the type and directionality of change vary greatly across scleractinians, partly because of the diversity of micromorphological shapes in corals (e.g., cerioid vs. meandroid vs. plocoid) and partly because local environmental factors differ between reefs and ocean basins. In a global meta-analysis of light scattering in coral colonies, Swain et al. (2018) found that calice structure had a larger influence on bleaching susceptibility than colony structure, highlighting the importance of corallite shape in coral biology.

Like morphology, coral physiology often shifts along a light gradient, primarily tied to the mixotrophic strategy of the coral. In high light environments, corals typically require fewer symbionts and photosynthetic pigments to achieve high photosynthate production (Roth 2014; Levy et al. 2016; Eyal et al. 2019). Thus, shallow corals are often associated with thicker tissues and higher macromolecule concentration (Roth 2014; Eyal et al. 2019; Martinez et al. 2020). Conversely, deep corals often store fewer macromolecules but are characterized by higher symbiont densities and higher concentrations of photosynthetic pigments to maximize photosynthetic capacity (Roth 2014; Eyal et al. 2019; Martinez et al. 2020).

Physiological shifts with light availability may also be accompanied by shifts in mixotrophic strategy along a light gradient (Muscatine et al. 1989; Alamaru et al. 2009; Fox et al. 2018). Corals in shallow, high light environments where photosynthetic production is greater may not require as much heterotrophic feeding as in deep environments, where lower photosynthetic potential necessitates compensatory heterotrophic feeding. Carbon and nitrogen isotope compositions are commonly used to quantify coral isotopic niche and infer degree of heterotrophy in corals (Muscatine et al. 1989; Conti-Jerpe et al. 2020; Chei et al. 2025). In corals, higher dependence on photosynthesis should result in lower δ^13^C and δ^15^N values that reflect direct sourcing of production from algal symbionts (Muscatine and Kaplan 1994; Einbinder et al. 2009). However, this pattern can be obscured by the sharing and recycling of metabolites, nutrients, and waste products between the symbiont and its coral host (Ferrier-Pagès and Leal 2019). Higher dependence on symbiotic production, for example in shallow corals with higher light availability, should result in stronger overlap between coral host and symbiont isotopic niches than in deep corals where divergence in isotopic niche is often inferred as enhanced heterotrophy (Conti-Jerpe et al. 2020; Sturaro et al. 2021).

Previous work has documented shifts in morphological, physiological, or trophic traits across light gradients; however, it remains unclear how some or all of these traits are leveraged synergistically across light environments, and whether responses vary across species or clades in a shared environment. We address this knowledge gap by quantifying a suite of morphological, physiological, and trophic traits along a light gradient in Puerto Rico. To ascertain the generality of these patterns among corals, we sampled six scleractinian species, with three each from the robust and complex clades. Because acclimatization and phenotypic plasticity are energetically costly to corals, we hypothesized that individual coral species may express a few specific biological strategies rather than all possible mechanisms simultaneously. Our results indicated that light strongly modulated the corallite morphology of all six species, but physiology and trophic strategy remained remarkably consistent and robust to changes in light availability. Trophic strategy differed between the clades, and we found that large calice species tended to show isotope patterns consistent with greater heterotrophy than species with smaller calices. These data are valuable for predicting how these foundational organisms will persist and function under changing light regimes driven by coastal development, climate change, and oceanographic shifts.

## Methods

### Field sampling

To test for morphological, physiological and trophic variation across light gradients, we collected corals along a light gradient in the center of the La Parguera Nature Preserve Marine Protected Area, Puerto Rico (17.935181°N, −67.048005°W) (Supporting Information Fig. S1). Reefs fringed the exposed sides of small cays approximately 5 km from the mainland. The reef sloped from 3 to 7 m deep over approximately 0.1 km then from 7 to 25 m deep over another 0.1 km. Below 25 m, hard substrate was replaced by loose fine silt and coral cover decreased rapidly. The site was exposed to moderate wave action and seasonal tropical storms, resulting in a relatively well-mixed water column. The waters off La Parguera are more turbid compared to many oceanic reefs around the world (Bejarano and Appeldoorn 2013), so attenuation of light happens rather quickly within the top 20 m of the water column. As such, our study system addresses steep light attenuation in turbid coastal systems, which represents an ecologically important and globally relevant light environment. As described in the results, the degree of change in light availability along the depth gradient was greater than the change in temperature or dissolved oxygen. Thus, we focused our analyses on the relationships between coral biology and light but acknowledge that there are other characteristics (e.g., water flow and food availability) of the environment that shift along the depth gradient and may affect the biology of these corals.

To quantify irradiance along the reef slope, we calculated the diffuse attenuation coefficient for downwelling irradiance (*K*_d[PAR]_ m^−1^) from 0 to 20 m. Photosynthetically Active Radiation (PAR, 400–700 nm) was measured using a cosine-corrected diving-pulse amplitude modulated sensor (Walz) which was previously calibrated against a manufacturer-calibrated quantum sensor (Li-1400, LI-COR). PAR measurements were collected by hand-carrying the sensor over the course of seven dives on three dates during the coral sampling period, at approximately 0.5 m depth intervals from 0.5 to 20 m below the surface. Observations from the three sampling dates were pooled and an exponential curve was fitted to the data based on the Beer–Lambert Law (Prada et al. 2022).

(1) $$E_{\text{z}}=E_{0}\times e^{K_{\text{d}(\text{PAR})}{\text{m}}^{-1}\times \text{z}}$$

where *z* is the depth and *E*_z_ is the irradiance at the given depth. Based on the fit of the curve, we calculated that the surface irradiance (*E*_0_) was 757 _*μ*_mol quanta m^−2^ s^−1^ and the *K*_d(PAR)_ m^−1^ was 0.187. Using these values, we calculated an irradiance value for each sample based on their collection depth which is used for all irradiance statistics throughout the manuscript.

To evaluate other environmental differences across the reef slope, we measured temperature and dissolved oxygen for 6 weeks leading up to sample collection. Sensors were deployed at the top (5 m) and bottom (18 m) of the reef slope on rebar hammered into sandy substrate. Every 10 min, a HOBO Pendant Temperature Logger (UA-002-64) recorded temperature and a HOBO Dissolved Oxygen Data Logger (U26-001, calibrated before deployment according to manual) recorded dissolved oxygen.

At each of four depths (4.5, 9.1, 13.7, and 16.8 m), we haphazardly collected fragments (approximately 1–10 cm^2^) from 10 different colonies of six species of scleractinian coral: *Montastraea cavernosa*, *Orbicella faveolata*, *Orbicella franksi*, *Agaricia agaricites*, *Porites porites*, and *Porites astreoides* (Supporting Information Fig. S2). *Montastraea cavernosa*, *O. faveolata*, and *O. franksi* are from the robust clade of scleractinians, while *A. agaricites*, *P. porites*, and *P. astreoides* are from the complex clade. Corals were only selected if they occurred at exposed areas along the slope that received direct light from the surface. Divers used a hammer and chisel to break off small fragments from the growing edge or a branch tip. Fragments were stored in whirl-pak bags with seawater and maintained in darkness until they were returned to the laboratory, cleaned of any visible debris or epiphytes, and frozen to −80°C using liquid nitrogen.

### Laboratory methods

Frozen samples were airbrushed to remove tissue from the coral skeleton using phosphate buffered saline (pH 7.4). The tissue slurry was homogenized and aliquoted for physiological assays and isotopic analysis. Symbionts were extracted from the host tissue as pellets via centrifugation at 13,000*g* for 3 min. The supernatant containing the host was evaluated under a microscope to confirm the absence of symbionts. All tissue slurries were maintained at −80°C until processing. Soluble protein was extracted from the host tissue using a Pierce Bicinchoninic Acid Protein Assay Kit (Cat # 23225, Thermo Scientific) with a bovine serum albumin standard according to the manufacturer instructions and was quantified on a plate reader (Agilent Synergy HTX Multi-Mode Reader) at a wave-length of 562 nm.

The concentration of chlorophyll *a* (Chl *a*), extracted from the symbionts in 100% acetone, was quantified via absorbance according to the microplate protocol from Warren (2008) using extinction coefficients from Jeffrey and Humphrey (1975). Symbiont density was quantified by resuspending symbionts in phosphate buffered saline (pH 7.4) and counting cells on a hemocytometer. Six to eight replicates of each sample were averaged, and samples were recounted if the coefficient of variation among replicates exceeded 15%. The ash free dry weight was quantified as a proxy for biomass for both the host and symbionts (Purcell 1996). To do this, separate aliquots of the host and symbionts were weighed after drying at 60°C for 24 h in pre-burned tin pans, then again after burning at 450°C for 4 h. The reported values were the difference between the dry and burned weights to represent only organic material. All physiological metrics were standardized to the homogenate volume after airbrushing and the total surface area of the skeleton, which was quantified using the wax dipping technique (Veal et al. 2010).

Morphological metrics were quantified using photographs of the bleached coral skeletons. After airbrushing, the coral skeletons were soaked in 10% bleach for 4 + h and then dried at 60°C for 2 + h. Each skeleton was photographed under stereomicroscopy with an iPhone camera with a ruler for scale. Here, we use “corallite” to refer to the entire skeletal area occupied by a single polyp (including surrounding coenosarc) and “calice” to refer to only the central, cup-shaped area in which the polyp sits (Supporting Information Fig. S3). We used imageJ to quantify the area, length, and width of five corallites and their calices per sample. Whenever possible, we avoided quantifying corallites on the growing edge of the fragment, as the morphology metrics could be affected by new growth. However, this was often unavoidable in *M. cavernosa* due to its large polyp size, which may have introduced some additional variation. The corallite and calice diameters are the mean of the length and width (Supporting Information Fig. S3). Corallite density is a count of all calices within an area of the skeleton measured in imageJ.

We conducted bulk stable carbon and nitrogen isotope analysis to quantify the isotopic niches of paired coral hosts and their symbionts. For a detailed description of the isotope quantification methods, see Supporting Information Text S1. Briefly, symbionts were removed from the host fraction via centrifugation, and the symbiont pellet was cleaned to ensure complete separation of the coral fractions. After freeze-drying, samples were packed into tin capsules and analyzed in an Elementar Vario Isotope Select Elemental Analyzer coupled with an Elementar Isoprime VisION Isotope Ratio Mass Spectrometer (EA-IRMS). Isotope values are presented in standard delta notation with units of permil (‰) relative to Vienna-Pee Dee Belemnite (δ^13^C) and atmospheric N_2_ (δ^15^N).

### Statistical methods

All statistics were computed in R (Version 2025.05.01 + 513). We conducted separate Student’s *t*-tests to quantify differences in temperature and dissolved oxygen parameters between the shallow (5 m) and deep (18 m) sites. Normality and homoscedasticity were assessed for each coral biology metric using Shapiro–Wilk and Bartlett’s tests, respectively. The data for most metrics were non-normal and heteroscedastic, and thus necessitated the use of nonparametric tests. We ran generalized linear models for each species to quantify shifts in corallite morphology, physiology, and isotope values across the predicted light gradient, using irradiance values calculated based on our *K*_d(PAR)_ m^−1^. Additionally, we used Spearman’s ranked correlation tests to quantify the relationships between each metric with irradiance for each species.

To characterize host-symbiont isotopic coupling of each coral species, we quantified the host and symbiont isotopic niches and calculated scores for the host’s reliance on symbionts. A full description of these methods can be found in Supporting Information Text S2. We used four metrics to assess differences between the isotopic niche of the symbionts and their host corals for each species. First, we created Bayesian models using Stable Isotope Bayesian Ellipses in R (SIBER) to compare the host and symbiont δ^13^C and δ^15^N values (Jackson et al. 2011). From this, we calculated the ellipse overlap between host and symbiont for each species. According to Conti-Jerpe et al. (2020), an ellipse overlap < 10% indicates heterotrophy, from 10% to 70% indicates mixotrophy, and > 70% indicates autotrophy. Second, we calculated the Euclidean distance between the centroids of host and symbiont ellipses for each species. We interpreted a centroid distance less than 1‰ as indicative of autotrophy, between 1‰ and 2‰ as mixotrophy, and greater than 2‰ as heterotrophy (Conti-Jerpe et al. 2020). Third, because the data were non-normal and heteroskedastic, we assessed the difference between host and symbiont isotope values using PERMANOVAs for each species. Following recent coral isotope studies (Fox et al. 2023; Travaglione et al. 2023; Chei et al. 2025), we interpret stronger host–symbiont overlap as greater isotopic coupling within the holobiont and lower overlap as greater isotopic decoupling, patterns that are consistent with differences in trophic strategy but can also be influenced by baseline isotope variation and internal nutrient recycling. Finally, we calculated the HERS index (Host Evaluation: Reliance on Symbionts) following a similar approach outlined by Pons et al. (2025), with on bootstrapping methods from Fox et al. (2023). The HERS index generates a score on a scale of 0 (complete host-symbiont decoupling) to 1 (complete host-symbiont coupling) (Chei et al. 2025; Pons et al. 2025).

To test for a shift in trophic strategy across the light gradient, we repeated SIBER and HERS analyses for each species at each of the four depth categories (4.5, 9.1, 13.7, and 16.8 m). For each species at each depth, we conducted PERMANOVAs and calculated the ellipse overlap, centroid distance, and HERS score as described above (Supporting Information Table S1). We then conducted Spearman’s rank correlations to compare each metric against calculated irradiance values for each species because of small sample sizes. Finally, all six species were pooled to quantify the relationship between ellipse overlap or centroid distance and our five morphology metrics, which were assessed using linear models.

## Results

### Light environment changes with depth

Photosynthetically active radiation decreased across the depth gradient (Fig. 1a). Based on our calculated irradiance curve, maximum daytime irradiance for the corals collected decreased one order of magnitude from 361.05 _*μ*_mol quanta m^−2^ s^−1^ at 3.96 m to 18.63 _*μ*_mol quanta m^−2^ s^−1^ at 19.81 m. Compared to the change in light across the depth gradient, temperature and dissolved oxygen did not exhibit ecologically meaningful shifts (Fig. 1b,c). Over the 6-week observation period, seawater temperature was only 0.02°C higher at the shallow site (29.39 ± 0.29°C) than the deep site (29.37 ± 0.27°C) (*t*[23,956], *t*-ratio = 4.61, *p* < 0.0001), and both sites were highly oxygenated (> 7 mg L^−1^) with dissolved oxygen slightly higher at the deep site (7.46 ± 0.34 mg L^−1^) than the shallow site (7.02 ± 0.07 mg L^−1^) (*t*[17,582], *t*-ratio = −62.54, *p* < 0.0001).

### Morphology and physiology across the light gradient

All six species exhibited shifts in their corallite morphology across the light gradient (Fig. 2; Supporting Information Table S2; Supporting Information Fig. S4). All species had significantly higher corallite density in high light environments (MCAV: *p* = 0.003, OFAV: *p* = 0.0009, OFRA: *p* = 0.0001, AAGA: *p* = 0.0004, PAST: *p* < 0.0001, PPOR: *p* < 0.0001). Five species (with the exception of *P. porites*) exhibited a negative relationship between light availability and corallite size, while corallite diameter (MCAV: *p* = 0.002, OFAV: *p* = 0.008, OFRA: *p* = 0.004, AAGA: *p* = 0.004, PPOR: *p* = 0.030) and area (MCAV: *p* = 0.005, OFAV: *p* = 0.002, OFRA: *p* = 0.0008, AAGA: *p* = 0.008, PPOR: *p* = 0.024) were significantly larger in darker environments. Only *M. cavernosa* exhibited morphological change in calice size, with significantly lower calice diameter (*p* = 0.007) and area (*p* = 0.037) in high light environments.

Overall, we observed physiological changes across the light gradient in only a few species (Fig. 2; Supporting Information Table S2; Supporting Information Fig. S5). Protein content was higher in high light environments in *O. franksi* (*p* = 0.040) and *P. porites* (*p* = 0.017), and host biomass was positively correlated with high light in *A. agaricites* (*p* = 0.035), *P. porites* (*p* = 0.028), and *P. astreoides* (*p* = 0.005). Symbiont biomass was negatively associated with light availability in *P. astreoides* (*p* = 0.046), and symbiont density was positively associated with light in *M. cavernosa* (*p* = 0.002). Chlorophyll *a* only changed significantly in *P. porites*, with a higher concentration of Chl *a* in high light environments (*p* = 0.039).

### Trophic patterns persist across the light gradient

The mean isotope composition varied among species (Supporting Information Fig. S6). Individual isotope values and C : N ratios in each species for both the host and symbiont fractions were generally consistent across the light gradient, with a few exceptions (Fig. 2; Supporting Information Table S2; Supporting Information Fig. S7). δ^13^C values of the host increased with light availability in *M. cavernosa* (*p* = 0.0003), *O. faveolata* (*p* = 0.005), and *P. porites* (*p* = 0.0001), while δ^13^C values of the symbionts only increased with light availability in *P. porites* (*p* < 0.0001). Host δ^15^N values did not change significantly with light availability, but symbiont δ^15^N values had a positive relationship with light in *A. agaricites* (*p* = 0.031) and *P. astreoides* (*p* = 0.037). C : N ratios did not change significantly with light availability in the host or symbiont for any of the six species.

SIBER and HERS analyses showed that in most species the coral host occupied an isotopic niche distinct from that of its symbionts, whereas *P. astreoides* showed substantially greater host–symbiont overlap (Fig. 3, Fig. 4, Table 1; PERMANOVAs: MCAV: *p* = 0.001, OFAV: *p* = 0.002, OFRA: *p* = 0.001, AAGA: *p* = 0.002, PPOR: *p* = 0.04, PAST: *p* = 0.86). Ellipse overlaps ranged from 0% to 60% (MCAV = 0%, OFAV = 2%, OFRA = 0%, AAGA = 23%, PPOR = 17%, PAST = 60%). Centroid distances for all six species were greater than 1‰ and were as high as 4.3‰ in *O. franksi* (MCAV = 2.97‰, OFAV = 3.27‰, OFRA = 4.30‰, AAGA = 2.66‰, PPOR = 1.33‰, PAST = 1.16‰). The six species exhibited a range of mean HERS scores from 0.27 to 0.76 (MCAV = 0.29 ± 0.08, OFAV = 0.41 ± 0.08, OFRA = 0.27 ± 0.08, AAGA = 0.63 ± 0.14, PPOR = 0.53 ± 0.16, PAST = 0.76 ± 0.10). Importantly, none of these host–symbiont coupling metrics changed significantly across the light gradient in any of the six species (Supporting Information Figs. S8 and S9).

The centroid distance (CD) and ellipse overlap (EO) of the host and symbiont isotopic niches shifted significantly with the morphology of each species (Fig. 5). Corals with greater overlap between their host and symbiont isotopic niches were associated with higher corallite density (CD: *R*^2^ = 0.51, *p* < 0.0001; EO: *R*^2^ = 0.29, *p* = 0.0064), lower calice diameter (CD: *R*^2^ = 0.38, *p* = 0.0012; EO: *R*^2^ = 0.28, *p* = 0.0076), and lower corallite area (CD: *R*^2^ = 0.29, *p* = 0.0062; EO: *R*^2^ = 0.24, *p* = 0.015) than corals with distinct host and symbiont isotopic niches. Calice diameter (CD: *R*^2^ = 0.075, *p* = 0.20; EO: *R*^2^ = 0.13, *p* = 0.081) and area (CD: *R*^2^ = 0.026, *p* = 0.46; EO: *R*^2^ = 0.083, *p* = 0.17) did not change with trophic strategy due to the large calice size of *M. cavernosa*. With *M. cavernosa* removed from the analysis, calice diameter (CD: *R*^2^ = 0.71, *p* < 0.0001; EO: *R*^2^ = 0.33, *p* = 0.0079) and area (CD: *R*^2^ = 0.71, *p* < 0.0001; EO: *R*^2^ = 0.31, *p* = 0.011) were both significantly smaller in corals with more isotopic overlap than those with distinct isotopic niches.

## Discussion

We quantified morphological, physiological, and trophic changes along a light gradient in six Caribbean scleractinian species, and explored possible linkages among them. We observed significant shifts in corallite morphology across the depth and light gradient, likely as plastic responses to maintain internal homeostasis. Despite these morphological shifts, all six coral species exhibited remarkably stable physiology and trophic strategies. Notably, each of the six species displayed one trophic strategy (either autotrophy, mixotrophy, or heterotrophy) along the full range of the light gradient, and each species’ trophic strategy aligned with their evolutionary clade (robust vs. complex). Physiological stability across the light gradient was associated with shifts in corallite morphology and with isotope patterns consistent with greater heterotrophic contribution to the holobiont. In particular, we found a link between skeletal morphology and trophic strategy in which large-corallite species tended to show more decoupled host–symbiont isotopic niches, consistent with greater heterotrophic contribution. These findings are consistent with the hypothesis that corals plastically change their morphology in response to changing light conditions to maintain physiological and trophic homeostasis.

### Trophic strategy in Caribbean corals

Isotope values of the host and symbiont shifted across the light gradient, with *M. cavernosa*, *O. faveolata*, and *P. porites* showing significant, positive correlations between the host tissue δ^13^C and irradiance (Fig. 2). This pattern suggests greater carbon isotope fractionation as carbon fixation declined under lower irradiances, a pattern seen in other scleractinian corals (Einbinder et al. 2009; Fox et al. 2018; Wall et al. 2020). In the symbionts, this pattern was conserved only in *P. porites*, indicating decoupling between symbiont and host carbon translocation in the other species. The δ^15^N values of the host did not exhibit differences along the light gradient in any of the six species, suggesting a similar strategy for nitrogen acquisition, regardless of light levels. Previous studies have found uniform patterns of δ^15^N values across a depth gradient in both the coral host and symbionts (Alamaru et al. 2009; Wall et al. 2020). However, in the symbionts of *P. astreoides* and *A. agaricites*, higher light environments were correlated with higher δ^15^N values. Coral-associated symbionts can also show relatively low δ^15^N values in low light due to lower growth and photosynthesis compared to high light counterparts (Muscatine and Kaplan 1994). The increase in *P. astreoides* aligns with a negative correlation between symbiont biomass and light availability, which may be explained by *P. astreoides*’ reliance on photosynthetically derived carbon. Taken together, we found that carbon and nitrogen isotope niche space of symbionts and hosts were more distinct from each other and less variable with light availability than previous studies (Alamaru et al. 2009; Fox et al. 2018; Wall et al. 2020).

Three other factors may influence isotope composition of corals along light gradients. First, differences in tissue composition and macromolecule storage with varying light availability could impact bulk tissue isotope values. For example, enhanced lipid storage would lower δ^13^C values because lipids have lower δ^13^C values relative to protein (DeNiro and Epstein 1977). While we observed shifts in host biomass and protein concentration in a few species, the C : N ratios of the hosts and symbionts remained similar along the light gradient in all six species, making it unlikely that the biochemical composition of the tissues strongly influenced isotope composition. Second, isotope values may reflect shifts in food or nutrients along a depth gradient. The isotope values of dissolved nutrients, POM, and zooplankton vary with depth due to oceanographic forces or shifts in primary productivity (Baker et al. 2011; Wyatt et al. 2012). We hypothesize that if this were the case, we would see more uniform responses to the light gradient across the six species, rather than discordant patterns and strengths of correlation among species. Thus, species-specific patterns suggest the isotope values observed here reflect patterns consistent with trophic strategy under these conditions. Finally, symbiont community composition in corals shifts over depth gradients (Bongaerts et al. 2013) and can affect coral symbiont physiology (e.g., symbiont biomass, density, chlorophyll) and trophic ecology (Wall et al. 2020). Our results indicated relatively stable physiology and isotope values in most species along the light gradient, meaning that symbiont communities would have to produce a compensatory response to light availability to produce these results. However, without identifying symbiont community composition, its impact on isotope values across depths cannot be ruled out.

The results of our SIBER and HERS analyses revealed generally low niche overlap between hosts and symbionts, reflecting substantial host–symbiont isotopic decoupling. In coral isotope studies, this pattern is commonly interpreted as consistent with greater heterotrophic contribution to the holobiont (Chei et al. 2025; Pons et al. 2025), although alternative processes including depth-related variation in resource isotope composition, internal nutrient recycling, and microbial reworking can also influence the degree of host–symbiont separation. Because we did not quantify POM or zooplankton isotope baselines across the depth gradient, we cannot fully partition those alternative explanations. However, the absence of significant depth-related shifts in ellipse overlap or centroid distance suggests that any baseline changes with depth were insufficient to generate a directional change in host–symbiont isotopic coupling within species in this dataset. While niche overlap is usually attributed to the transfer of autotrophically derived metabolites from the symbiont to the host, there are other possible phenomena that explain the degree of overlap between coral and symbiont niches. Heterotrophically derived metabolites may be reverse translocated to the symbionts, especially in low symbiont density corals such as temperate corals (Lindsay et al. 2025). Additionally, nitrogenous waste sharing between the symbiont and host would bring their isotopic niches closer together, increasing niche overlap and giving the impression of autotrophy (Chei et al. 2025; Pons et al. 2025). Microbial reworking of metabolites within the host may further cause isotope variation, complicating niche overlap interpretation. In our high light, tropical system, greater isotopic niche overlap is most parsimoniously interpreted as greater autotrophic coupling, although disentangling this from waste sharing or microbial reworking will require additional work (McCarthy et al. 2007; Ferrier-Pagès and Leal 2019).

The six species of coral had a notable split in trophic strategy by scleractinian clade which was retained across the light gradient. *Montastraea cavernosa*, *O. faveolata*, and *O. franksi*, which are all part of the robust (Vacatina) suborder, had distinct host and symbiont isotopic niches, indicating heterotrophy. Conversely, *P. porites* and *A. agaricites*, which are both from the complex (Refertinia) suborder, had mixed autotrophy and heterotrophy indicators, while *P. astreoides* was primarily influenced by autotrophy. Previous SIBER analyses in corals show that various trophic strategies can be found within the same environment. In Hong Kong, Conti-Jerpe et al. (2020) found two autotrophs, two mixotrophs, and three heterotrophs all living in the same local environment. In Taiwan, Sturaro et al. (2021) identified one autotroph, three mixotrophs, and one heterotroph living on the same reef. Of the 13 genera analyzed between these two studies and our study, trophic strategy generally divides across clades, with most complex corals classified as autotrophic or mixotrophic and most robust corals classified as heterotrophic. Despite this phylogenetic control over trophic strategy, some coral genera appear to retain trophic plasticity (Price et al. 2021). In particular, different species of *Porites* have been classified as autotrophs, mixotrophs, and heterotrophs (Conti-Jerpe et al. 2020; Sturaro et al. 2021; Travaglione et al. 2023).

Despite the split in phylogenetic evolutionary history, the two scleractinian clades do not differ greatly in their biology, and to date only a few biological characteristics resolve the two groups (Ying et al. 2018). Initially, the robust/complex names were given based on observed differences in colony morphology and calcification between clades, with complex corals often creating branching, columnar or foliose colonies with lower density skeletons, while robust corals tend to be simpler structures and have high-density skeletons (Romano and Palumbi 1996). However, as genetics has refined coral phylogeny, these generalizations no longer resolve the two clades. Thus, it is challenging to ascertain why trophic strategy so often differs between the two clades.

### Physiological stability along light gradients

Physiological differences along the light gradient were minimal overall. In a few species, macromolecule storage followed expected trends, with corals at high light sites experiencing higher productivity, resulting in thicker tissues and greater macromolecule storage than their counterparts in dark environments (Roth 2014; Eyal et al. 2019; Martinez et al. 2020). However, host biomass and protein storage remained largely stable across species and light levels, suggesting consistent energy acquisition and metabolite usage along the gradient. An exception was *A. agaricites*, which exhibited an unexpected negative relationship between light and biomass. C : N ratios were stable in all six species across the light gradient, indicating no significant reorganization of macromolecule storage (e.g., protein vs. lipid storage). We hypothesize that this physiological stability was largely sustained by the greater inferred heterotrophic contribution across these species.

In low light environments, corals typically increase symbiont density and pigmentation to enhance light harvesting via photosynthesis (Roth 2014; Levy et al. 2016; Eyal et al. 2019). The higher Chl *a* concentration in *P. porites* in high light environments is likely the result of higher polyp density, protein concentration, and host biomass. Here, we propose that the strong influence of heterotrophy in Caribbean corals decouples their biology from light availability, leading to minimal physiological changes across light gradients. An extreme example of this phenomenon occurs in temperate corals, which maintain symbiont populations even when light is scarce and their nutrition is primarily heterotrophic (Lindsay et al. 2025). When corals can sustain themselves primarily through heterotrophy, photosynthetic investments (such as pigments and symbionts) become less essential and may only be maintained in abundant light. This is supported by the negative relationship between light and symbiont biomass observed in *P. astreoides*, the only species classified as primarily autotrophic in our study. Thus, we found that higher dependence on autotrophy was associated with expected patterns of physiology along a light gradient.

### Shifts in corallite morphology along light gradients

We documented shifts in corallite morphology along the light gradient in all six species, with corallite density increasing in high light environments. This pattern is common across ocean basins, colony morphologies, and trophic strategies, although exceptions exist (Todd et al. 2004; Einbinder et al. 2009; Studivan et al. 2019). Our findings align with three key micromorphological responses observed in corals along light gradients, each closely tied to their energy acquisition strategy. First, in light-limited environments, corals increase internal light scattering through adjustments in corallite density, corallite/calice size, and calice relief, all of which serve to maximize light harvesting by symbionts (Todd 2008; Soto et al. 2018; Swain et al. 2018). Second, excessive light can be harmful to coral photosystems, leading to photoinhibition (Winters et al. 2003; Lesser 2024). To mitigate this, corals employ self-shading strategies at both the colony and corallite levels, reducing light absorption (Enríquez et al. 2005; Kaniewska et al. 2011). Third, corallite shape and size are directly linked to polyp size, which influences feeding capacity, although there is mixed evidence on whether or not larger polyps are associated with changes in prey type or feeding rate (Porter 1976; Sebens et al. 1996; Palardy et al. 2005). Self-shading and light harvesting work through the same morphological mechanisms but in response to opposite ends of the light gradient, suggesting a unified morphological toolkit for managing light stress. The influence of calice size on heterotrophy, however, is less straightforward. Unlike light harvesting and self-shading, heterotrophic capacity is not necessarily tied to light availability and may follow a different response along the light gradient.

Recent work suggests that calice relief, rather than calice size, is critical to light scattering. Corals transplanted from low to high light environments developed deeper calices with steep walls (Ow and Todd 2010), while high to low light transplants developed an open structure with shallow walls. In the wild, coral calices are commonly deeper (greater relief) in high light environments (Soto et al. 2018). Regardless of calice size, deeper calices decrease the amount of light reflected back up to the symbionts while flat calices have a high capacity to reflect light. In shallow, high light habitats, this is likely a self-shading strategy used to prevent photoinhibition, while in deep areas, corals use reflected light to maximize autotrophy. While we did not quantify calice depth, we hypothesize that the smaller calices we observed in high light environments are characterized by greater relief than those in the low light environment.

### Morphology and trophic strategy covary across species

Our results indicate that morphology covaries with trophic strategy in scleractinian corals. We show that across multiple species, corallite density was negatively associated with SIBER centroid distance and positively associated with ellipse overlap, indicating that more autotrophic species had greater corallite density than more heterotrophic species (Fig. 5). In our dataset, species classified as more autotrophic (greater host-symbiont coupling) tended to have smaller corallites than species classified as more heterotrophic (weaker host-symbiont coupling). This pattern corroborates findings by Conti-Jerpe et al. (2020), who found a relationship between corallite size and trophic strategy in seven species of Pacific corals. They proposed that smaller polyps increase the surface area to volume ratio of the corals, which has previously been associated with autotrophy in corals (Porter 1976; Jackson et al. 1985). As discussed above, larger calices should have a higher capacity for food capture, but there is mixed evidence for (Porter 1976) and against (Sebens et al. 1996; Palardy et al. 2005) this hypothesis. Despite the lack of direct evidence for increased feeding, large-caliced species tended to show isotope patterns consistent with greater heterotrophic contribution. This pattern is also consistent with the clade-level split in trophic strategy. Robust corals typically have larger calices and greater heterotrophy than their relatives in the complex clade. It remains unclear whether robust corals evolved larger calices, conferring a higher capacity for heterotrophy, or if calice size covaries with another trait that influences heterotrophy (e.g., symbiont fidelity and stress tolerance). Disentangling these evolutionary mechanisms requires further examination.

This study synthesized biological strategies across light gradients in Caribbean corals by quantifying trophic ecology, physiology, and morphology in multiple species. Overall, the trophic strategy of most species appeared to be highly influenced by heterotrophy, with a notable split in trophic strategy between clades. Light availability was strongly associated with a shift in corallite morphology across genera, potentially contributing to physiological stability across the light gradient. Finally, we provide evidence of a relationship between trophic strategy and corallite morphology, although shared evolutionary history and correlated trait evolution may account for this pattern as much as local environmental conditions. In doing so, our work highlights how environmental heterogeneity in nearshore reefs can drive meaningful phenotypic differentiation. This underscores the broader ecological relevance of our findings to coral populations living in turbid, coastal reef environments worldwide, which are increasingly common under contemporary coastal development and climate-driven change (Zweifler et al. 2021). Although our work did not encompass mesophotic corals, our findings highlight the potential for heterotrophic, low light corals to sustain physiological function compared to their high light counterparts without the associated thermal and irradiance stress. In the face of rapid environmental change, it is critical to understand the capacity of low light corals, such as mesophotic, turbid, and temperate corals, to support reef ecosystem function.

## Supplementary Material

Supplement1

Additional Supporting Information may be found in the online version of this article.

## Figures and Tables

**Fig. 1.**
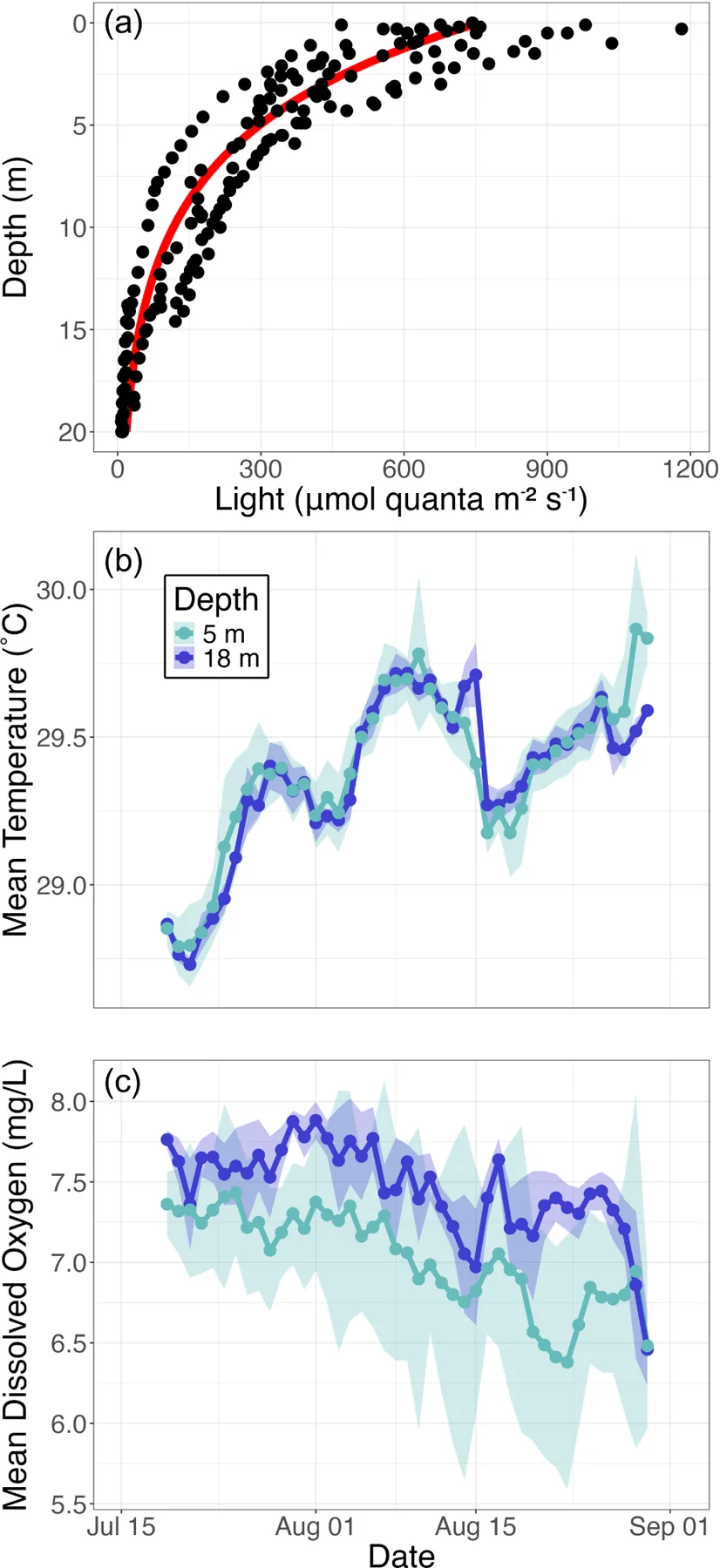
Light, temperature, and dissolved oxygen were quantified across depths. (**a**) The exponential light attenuation, based on a *K*_d(PAR)_ m ^1^ of 0.187, along the reef slope is indicated by the red line. Black dots indicate individual values collected using a Diving-PAM. For both (**b**) mean daily temperature and (**c**) mean daily dissolved oxygen, dots indicate daily means, and shaded regions indicate daily standard deviation.

**Fig. 2.**
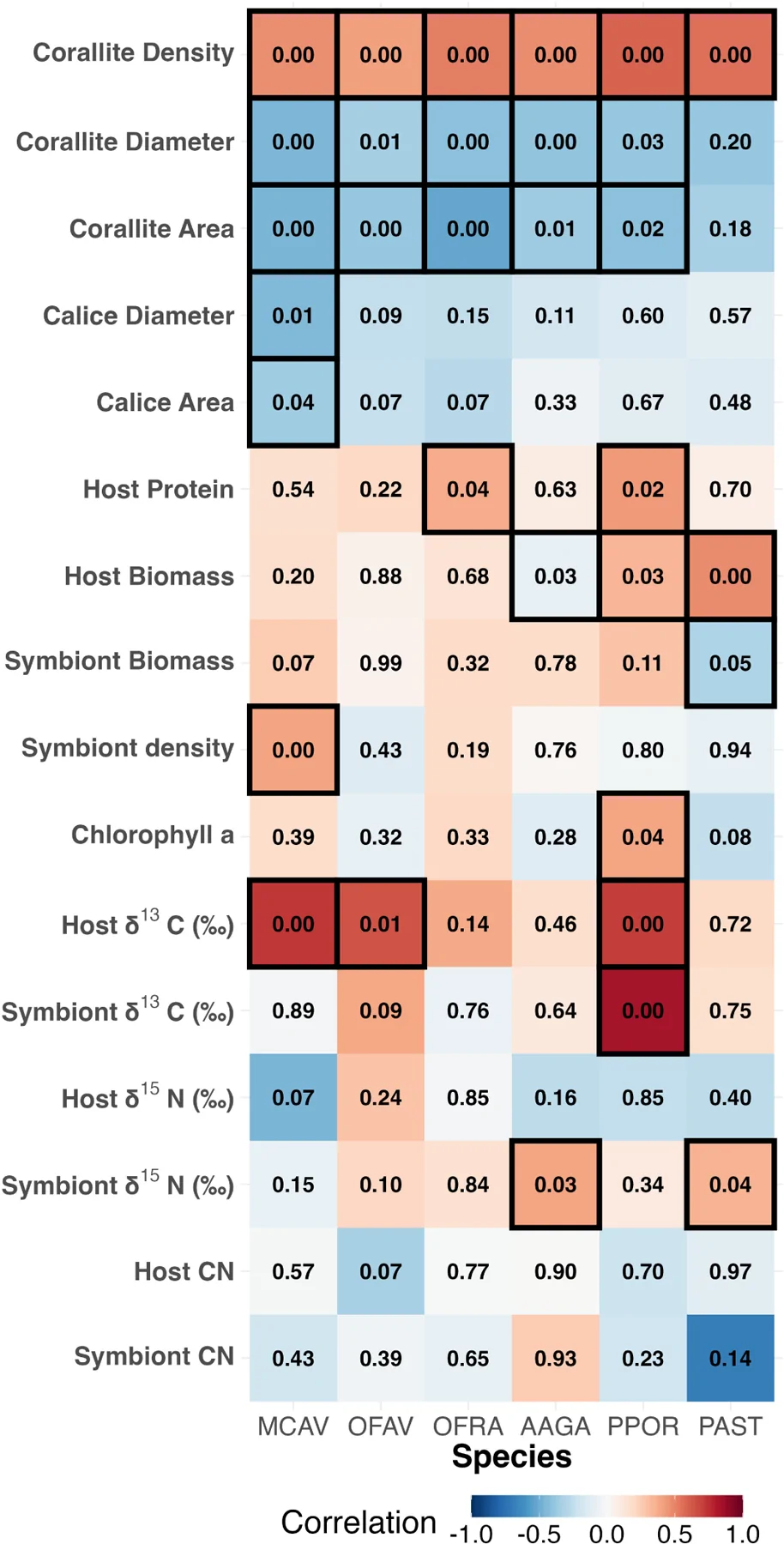
Correlation heatmap between light availability and corallite morphology, physiology and bulk isotope metrics. Box colors represent Spearman’s Rank Correlation between each variable and irradiance at the collection depth calculated using our *K*_d_. Each box contains the *p*-value from the generalized linear model for each species, with black borders around significant values (*p* < 0.05).

**Fig. 3.**
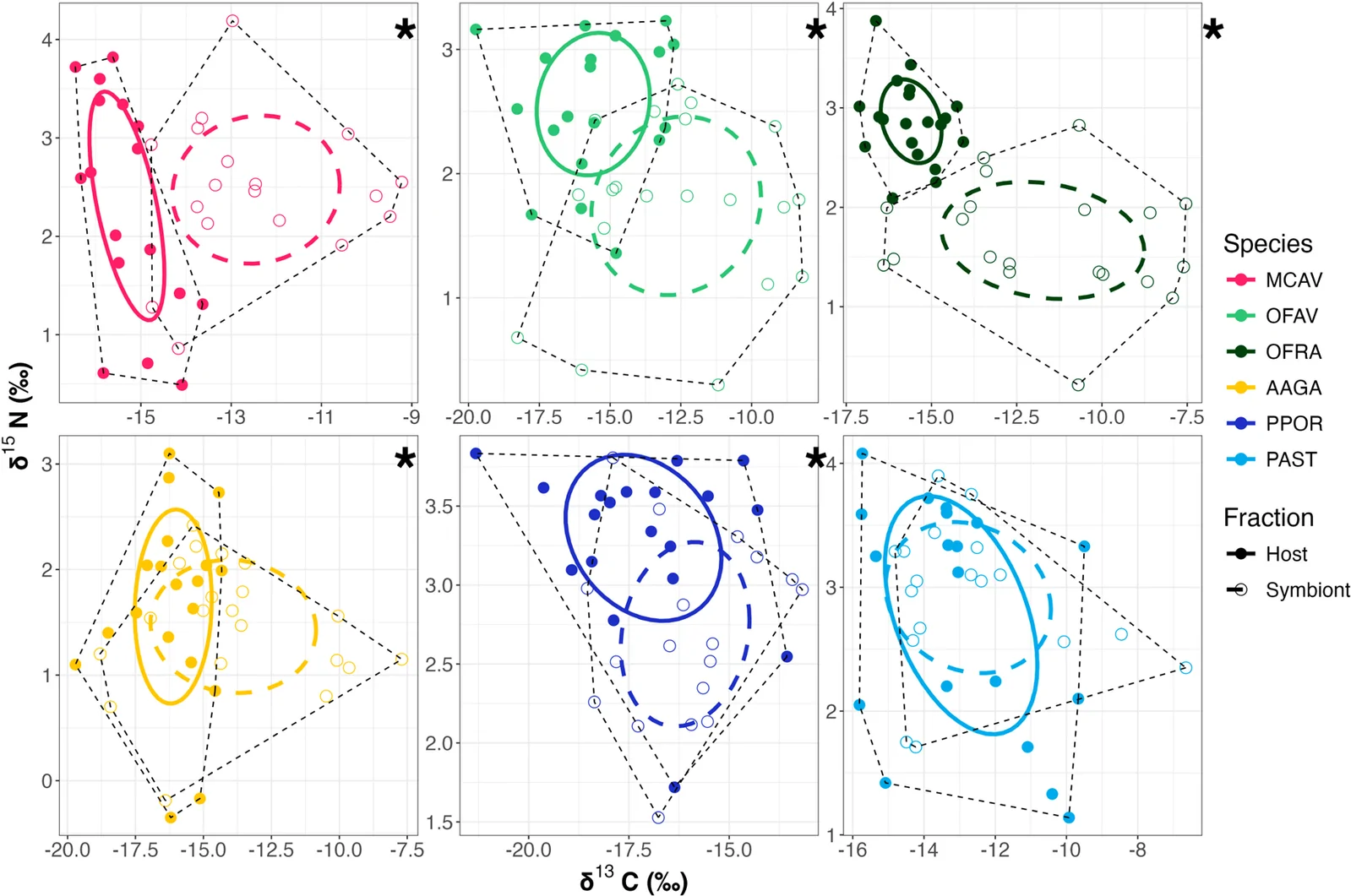
Isotope biplots of the relationship between the coral host (filled dots, solid line) and its symbionts (empty dots, dotted line) for six Caribbean species, with all depths pooled. Black dotted lines illustrate the convex hulls of each group, indicating the total area of each isotopic niche. Ellipses represent the 40% confidence interval of each isotopic niche based on SIBER analysis. Stars indicate host and symbiont pairs that occupy significantly different isotopic niches according to our PERMANOVAs (Table 1).

**Fig. 4.**
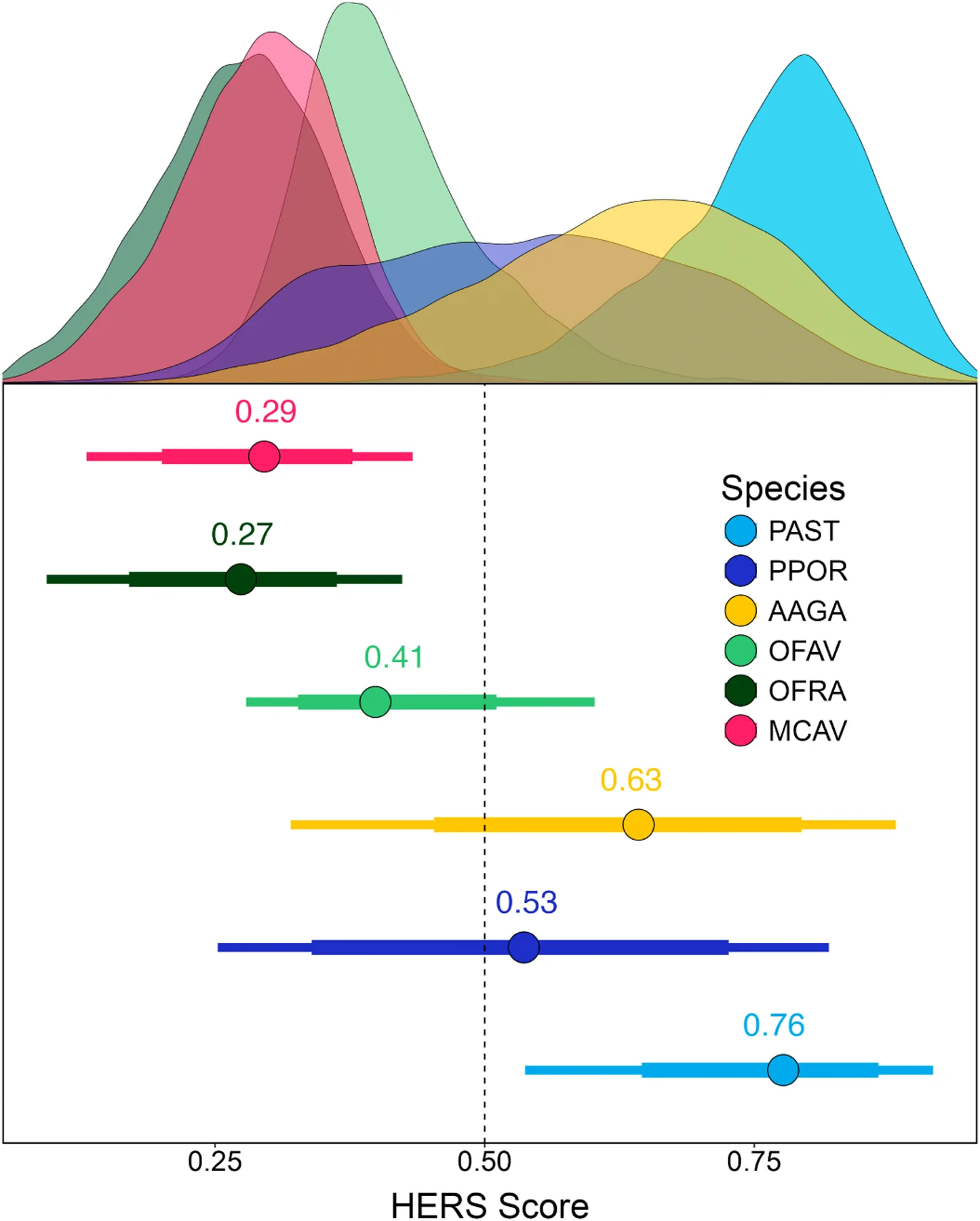
Host evaluation: reliance on symbionts (HERS) scores for each species with all depths pooled show that *M. cavernosa*, *O. franksi*, and *O. faveolata* are more heterotrophic, while *A. agaricites* and *P. porites* are intermediate mixotrophs, and *P. astreoides* is the most autotrophic. Dots indicate the mean HERS score ± 75% (thick bar) and 95% (thin bar) confidence intervals. The distribution of the 10,000 bootstrapped HERS score estimates is shown along the top.

**Fig. 5.**
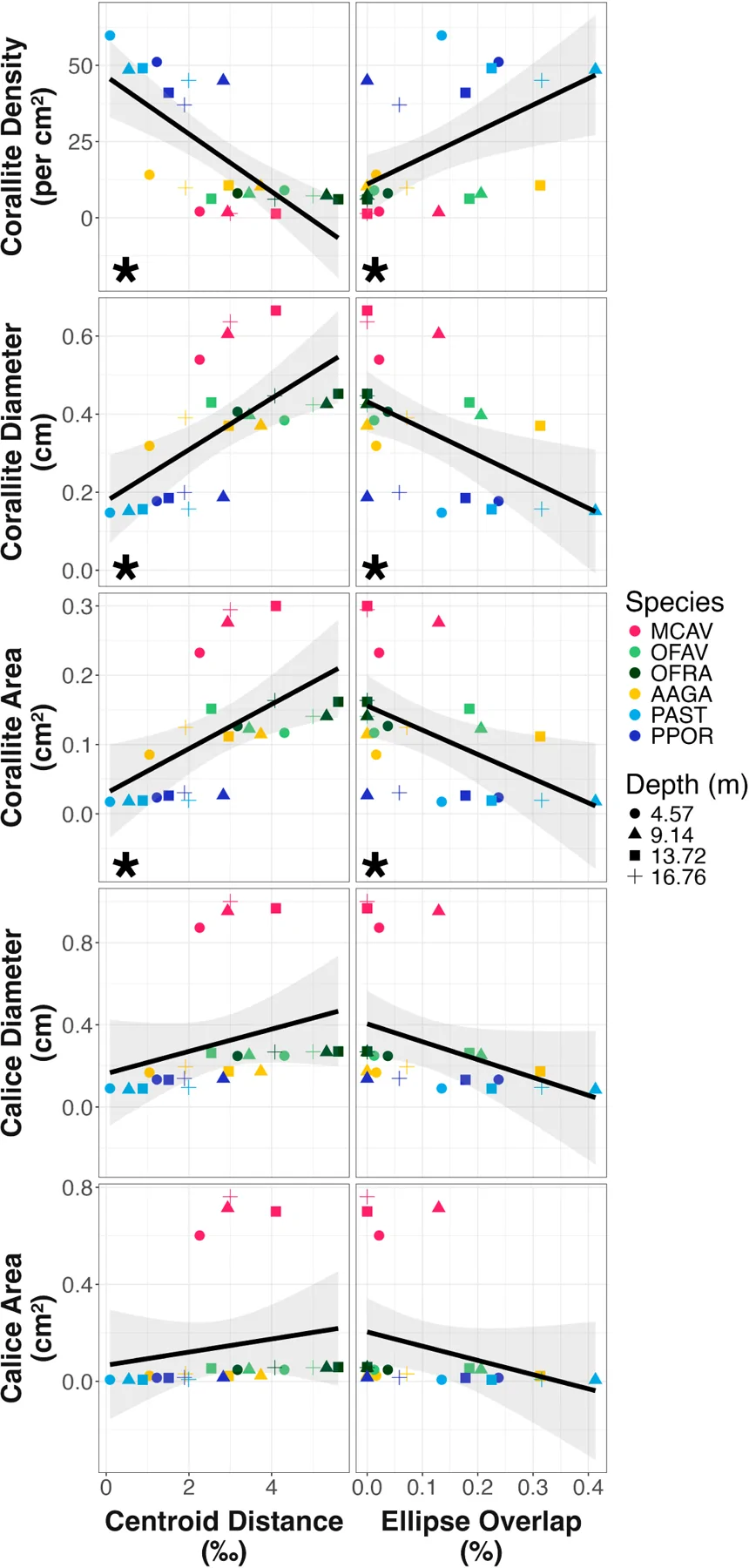
Morphology varies with trophic ecology. Individual values represent the centroid distance or overlap and the mean morphological value for each species at each depth. Summary statistics of each linear model are displayed within each graph. Black stars indicate a significant linear relationship (*p* < 0.05). Dots indicate values for each species in each depth bin, lines indicate the linear regression, and gray shading indicates a 95% confidence interval.

**Table 1.** Isotope values, SIBER and PERMANOVA results for six species of scleractinian coral. For the coral host and their symbionts, the mean δ^13^C and δ^15^N values are presented with their standard deviations. The total area (TA), corrected standard ellipse area (SEA_c_), Bayesian standard ellipse area (SEA_b_) and SEA_b_ 95% confidence interval are summarized for the symbionts and host. Additionally, a summary of differences between the symbiont and host includes the distance between the N and C means, the ellipse overlap (EO) and the centroid distance (CD). Finally, individual PERMANOVAs for each species describe differences between the symbiont and host isotopes, including the sum of squares (SS). The degrees of freedom for all six PERMANOVAs is 1. Significant values are in bold with a star.

Host						
Species	Mean δ^13^C (‰)	Mean δ^15^N (‰)	TA	SEA_c_	SEA_b_	SEA_b_ 95%
MCAV	−15.31 ± 0.81	2.31 ± 1.13	5.69	2.47	2.29	1.4–3.83
OFAV	−15.60 ± 1.95	2.56 ± 0.56	8.99	3.61	3.34	2.06–5.29
OFRA	−15.60 ± 0.87	2.87 ± 0.41	3.29	1.15	1.05	0.66–1.66
AAGA	−16.11 ± 1.38	1.65 ± 0.90	11.24	4.12	3.79	2.3–5.93
PPOR	−17.14 ± 1.90	3.30 ± 0.51	9.18	3.14	2.81	1.84–4.64
PAST	−12.96 ± 2.09	2.77 ± 0.94	14.59	5.91	5.41	3.38–8.7
Symbionts						
Species	Mean δ^13^C (‰)	Mean δ^15^N (‰)	TA	SEA_c_	SEA_b_	SEA_b_ 95%
MCAV	−12.45 ± 1.81	2.47 ± 0.73	10.59	4.42	3.97	2.52–6.50
OFAV	−12.67 ± 2.93	1.74 ± 0.70	17.31	6.78	6.14	3.94–9.81
OFRA	−11.73 ± 2.90	1.67 ± 0.57	14.62	5.44	4.94	3.15–7.82
AAGA	−13.90 ± 2.98	1.46 ± 0.62	14.93	6.07	5.51	3.41–8.72
PPOR	−16.10 ± 1.57	2.69 ± 0.57	6.74	2.94	2.65	1.65–4.34
PAST	−12.76 ± 2.26	2.92 ± 0.59	9.62	4.44	4.03	2.42–6.56

Symbiont vs. host				
Species	C distance	N distance	EO	CD
MCAV	2.86	0.16	0%	2.97
OFAV	2.93	0.82	2%	3.27
OFRA	3.87	1.2	0%	4.3
AAGA	2.21	0.19	23%	2.66
PPOR	1.04	0.61	17%	1.33
PAST	0.2	0.14	60%	1.16
PERMANOVA results				HERS index
SS	*R* ^2^	*F*	*p*	Score
71.78	0.43	24.79	0.001*	0.29 ± 0.08
90.31	0.27	13.58	0.002*	0.41 ± 0.08
163.85	0.47	33.85	0.001*	0.27 ± 0.08
47.78	0.18	7.88	0.002*	0.63 ± 0.14
13.45	0.1	4.03	0.04*	0.53 ± 0.16
0.56	0	0.1	0.86	0.76 ± 0.10

## Data Availability

All data, analysis, and visualization methods are publicly available on https://zenodo.org/records/21192369.
