# Supplementary material for: Morphological and trophic coupling distinguishes coral clades along a light gradient
Source: Limnol Oceanogr. Author manuscript; Available in PMC 2026 Nov 7. (PMC13741250; doi:10.1002/lno.70469)
Supplement: Supplementary file 1 [file NIHMS2218596-supplement-Supplement1.pdf]

Supplemental Materials for: Morphological and trophic coupling distinguishes coral clades along a light gradient

Taylor R. Lindsay[1§*]; Hana-lei Evans[2]; Morgan Schwartz[3]; Kelton W. McMahon[4]; Carlos Prada[1]

[1] University of Rhode Island, Center for Biotechnology and Life Sciences 120 Flagg Rd, Kingston, RI 02881

[2] James Cook University, College of Science and Engineering, 1 James Cook Drive, Douglas, QLD, 4814, Australia

[3] U.S. EPA, Atlantic Coastal Environmental Sciences Division, 27 Tarzwell Dr. Narragansett, RI 02882

[4] Graduate School of Oceanography, University of Rhode Island Coastal Resources Center, 220 S Ferry Rd, Narragansett, RI 02882

[§] Tufts University, Department of Biology, 200 College Ave, Medford, MA 02148

*Corresponding Author. Email: taylor.lindsay@tufts.edu

Bulk Isotope Methodology

We conducted bulk stable carbon and nitrogen isotope analysis to quantify the isotopic niches of paired coral hosts and their symbionts. Symbionts were removed from the host fraction via centrifugation at 1500 g for 3 min. The supernatant containing the host fraction was decanted and the symbiont pellet was washed with 1 mL PBS two times, reserving the supernatant each time for the host sample (Muscatine et al. 1989; Wall et al. 2020). The host slurry was evaluated under a microscope to confirm the absence of symbionts. Aliquots of separated host and symbiont were freeze-dried using a labconco freeze-dryer. 1.0 ± 0.1 mg of each fraction was packed into tin capsules and analyzed in an Elementar Vario Isotope Select Elemental Analyzer coupled with an Elementar Isoprime VisION Isotope Ratio Mass Spectrometer (EA-IRMS) at the U.S. EPA Atlantic Coastal Environmental Sciences Division, Narragansett, RI, USA. Isotope values are presented in standard delta notation with units of permil (‰) relative to Vienna-Pee Dee Belemnite ($\delta^{13}C$) and atmospheric N_2 ($\delta^{15}N$). Reference material USGS40 (L-glutamic Acid: %C = 40.8%, %N = 9.52%) and USGS41a (L-glutamic Acid: $\delta^{13}C$ = +36.55‰ ± 0.08, $\delta^{15}N$ = +47.55‰ ± 0.15) were used as certified quality control standards, and BLUM (blue mussel homogenate: $\delta^{13}C$ = -18.33‰ ± 0.27, $\delta^{15}N$ = 11.22‰ ± 0.38) and NIST 1547 (peach leaves: $\delta^{13}C$ = -25.89‰ ± 0.13, $\delta^{15}N$ = + 1.89‰ ± 0.15) were used as internal working standards. Results were only accepted if standards were within ± 0.5‰ of their accepted values. Standard deviations of replicate measurements of the standards over three batches were ≤ 0.06‰ for $\delta^{13}C$ and ≤ 0.15‰ for $\delta^{15}N$ (Balint et al. 2024). C:N ratios were determined from the EA-IRMS data as percentage of dry mass. The %N and %C were calculated by comparing the peak area of the samples to an internal standard curve of peak area. This was then compared to the standard reference materials cystine (%C = 29.98%, %N = 11.67%), USGS 40 (%C = 40.8%, %N =

9.52%) and acetanilide (%C = 71.09%, %N = 10.36%), which were used as our quality control standards. From this, the mg N and mg C were calculated for each sample, and then multiplied by the mass of the sample.

Bulk isotope statistical methods

To quantify the isotopic niches of the coral hosts and their symbionts, we created a Bayesian statistical model using Stable Isotope Bayesian Ellipses in R (SIBER) to compare the host and symbiont $\delta^{13}C$ and $\delta^{15}N$ values (Jackson et al. 2011). First, we calculated the standard ellipse areas through corrected (SEA_c) and Bayesian (SEA_b) approaches, containing 40% of the variation in each group. The Bayesian model iteratively fits multivariate normal distributions of each dataset using Monte Carlo simulations, yielding a probability density function with posterior estimates. Our models ran two chains of 10,000 interactions, burning the first 1,000 draws and thinning every 10 draws. We used four metrics to assess differences between the isotopic niche of the symbionts and their host corals for each species. First, from the SIBER analysis, we calculated the ellipse overlap between host and symbiont for each species. Greater overlap between ellipses was interpreted as more similar isotopic niches, and therefore, a greater production of carbon by photosynthesis. According to Conti-Jerpe et al. (2020), an ellipse overlap <10% indicates heterotrophy, from 10% to 70% indicates mixotrophy, and >70% indicates autotrophy. Second, we calculated the Euclidean distance between the centroids of host and symbiont ellipses for each species. We interpreted a centroid distance less than 1‰ as indicative of autotrophy, between 1‰ and 2‰ as mixotrophy, and greater than 2‰ as heterotrophy (Conti-Jerpe et al. 2020). Third, because the data were non-normal and heteroskedastic, we assessed the difference between host and symbiont isotope values using PERMANOVAs with 999 permutations for each species. Ellipse pairs that differed significantly

were deemed to rely on heterotrophy for their trophic ecology (Conti-Jerpe et al. 2020). Finally, we generated 10,000 bootstrapped estimates of the 95% and 40% ellipse overlaps in SIBER following Fox et al. (2023). From these estimates, we calculated the HERS index (Host Evaluation: Reliance on Symbionts) following a similar approach outlined by Pons et al. (Pons et al. 2025):

$$HERS = \frac{\left(SEO_H/SEA_H\right)^{exp\left(-SEO_S/SEA_S\right)} + \left(MEO_H/MEA_H\right)^{exp\left(-MEO_S/MEA_S\right)}}{2} \qquad \text{Eq 2}$$

where SEA and MEA represent the corrected standard ellipse areas (SEA_c) of the 40% and 95% ellipses, respectively, and the subscripts represent the host (H) or symbiont (S) fraction. The HERS index generates a score on a scale of 0 to 1, with 0 representing minimal overlap between the coral host and its symbionts (interpreted as heterotrophy) and 1 representing broad overlap (interpreted as autotrophy) in corals (Chei et al. 2025; Pons et al. 2025).

To test for a shift in trophic strategy across the light gradient, we repeated SIBER and HERS analyses for each species at each of the four depth categories (4.5 m, 9.1 m, 13.7 m, and 16.8 m). From each comparison, we first conducted PERMANOVAs to determine differences between the host and symbiont (Table S1). Then, we extracted the ellipse overlap and centroid distance between the host and its symbionts, as well as a HERS score. We then conducted Spearman's rank correlations to compare each metric against calculated irradiance values for each species because of small sample sizes. Finally, all six species were pooled to quantify the relationship between ellipse overlap or centroid distance and our five morphology metrics, which were assessed using linear models.

Additional References

Balint, S., Schwartz, M., Fowler, D. N., Linnekogel, S., Clemons, S. C., & Burkemper, L. K. (2024). Experimental assessment of elemental analyzer isotope ratio mass spectrometry normalization methodologies for environmental stable isotopes. *Rapid Communications in Mass Spectrometry*, *38*(17), e9837. https://doi.org/10.1002/rcm.9837

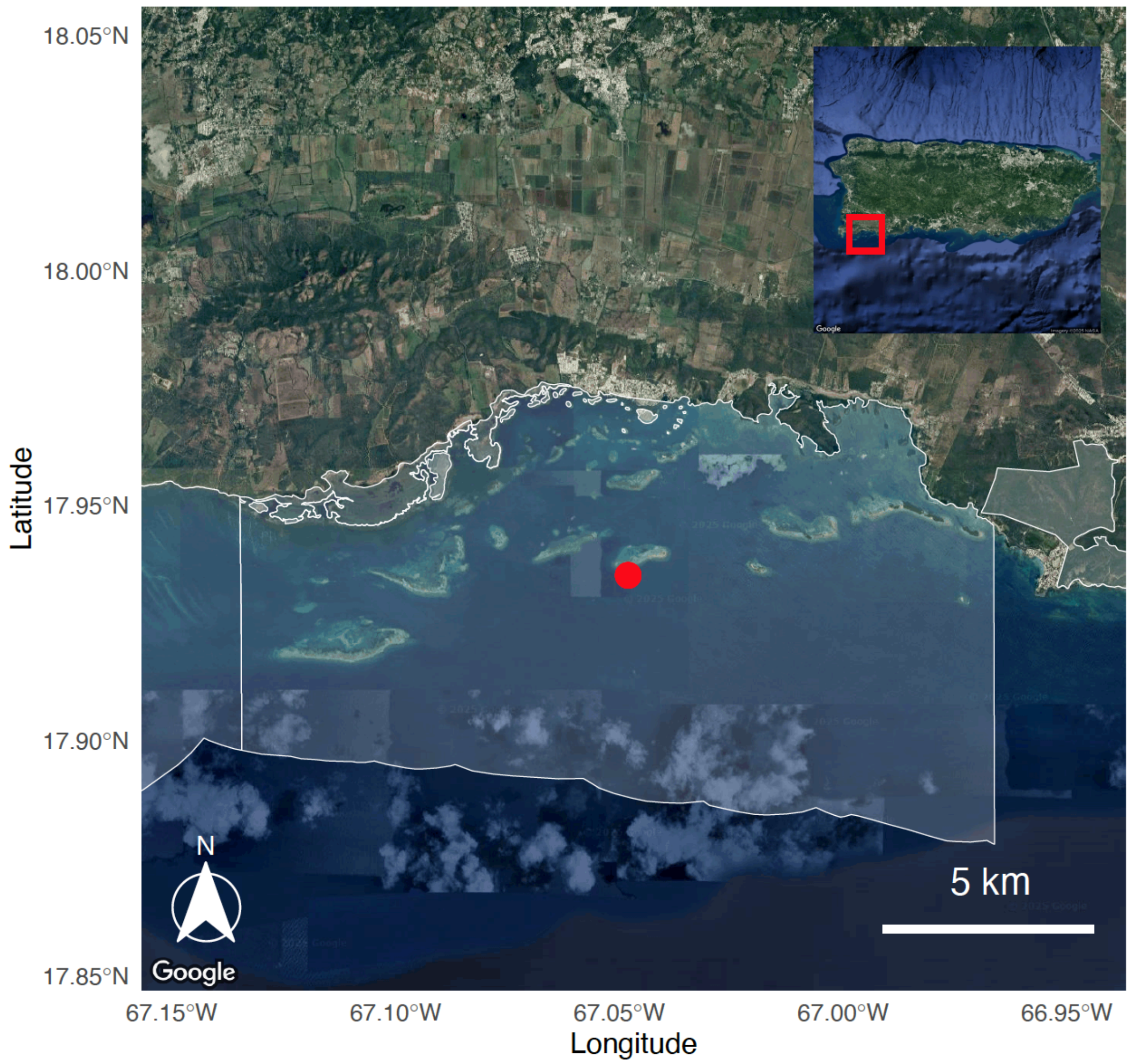

Figure S1. The sampling site in Puerto Rico is indicated by a red dot. White borders indicate marine protected areas. Our study is located within the La Parguera Nature Preserve.

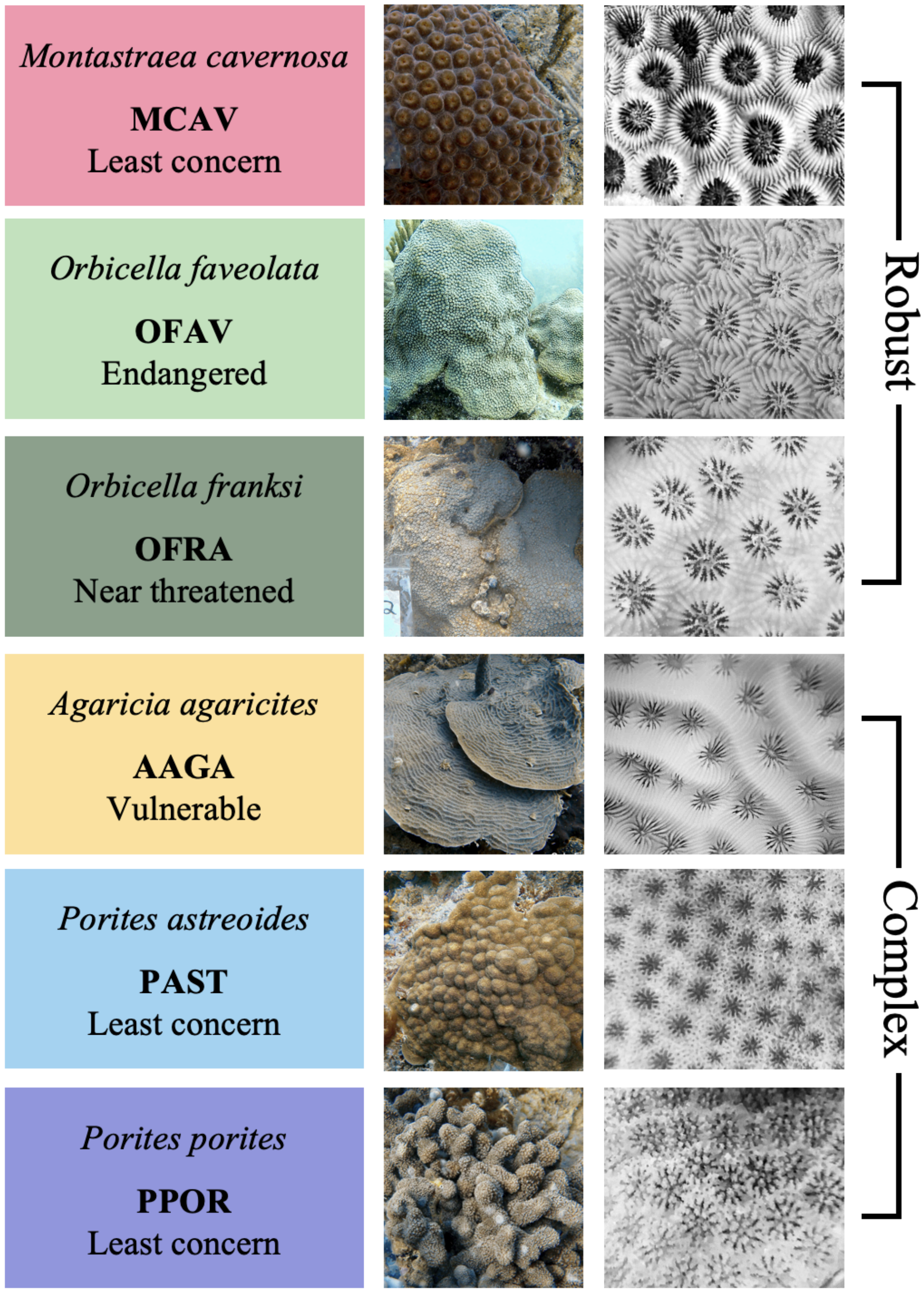

Figure S2. Species of coral collected from Puerto Rico with their abbreviations and ICUN Red List classifications. Species are divided into the robust and complex clades.

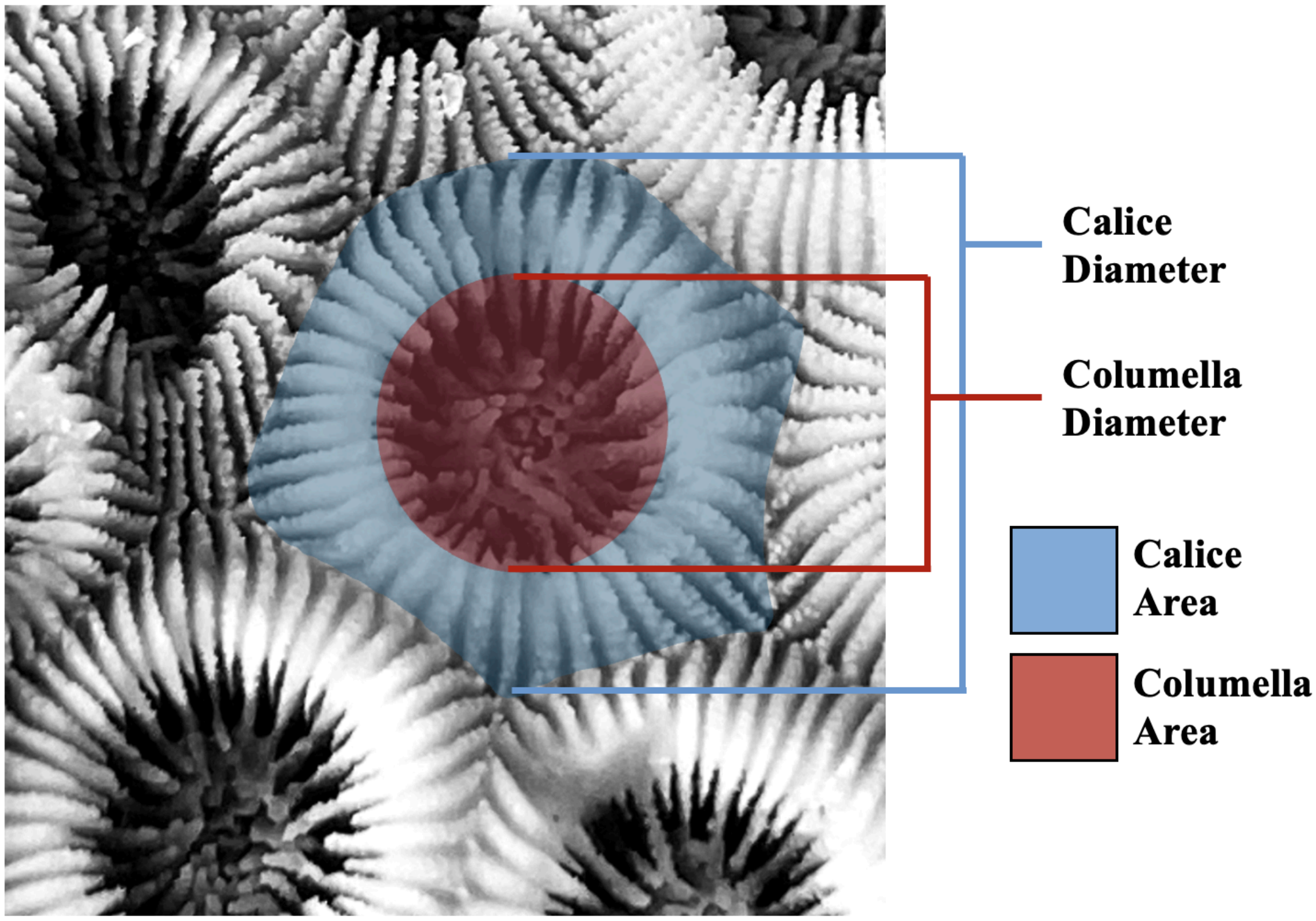

Figure S3. Illustration of calice morphometrics. The gray area represents the calice while the dotted area indicates the entire corallite.

Table S1. PERMANOVA results for six species of scleractinian coral separated by depth describe differences between the symbiont and host isotopes, including the sum of squares (SS). All PERMANOVAs had 1 degree of freedom. Significant values are in bold with a star.

Species	Depth	SS	R^2	F	*p*
MCAV	4.57	6.23	0.28	2.31	0.12
	9.14	11.62	0.32	2.84	0.14
	13.72	48.66	0.73	18.55	**0.007***
	16.76	16.71	0.64	14.28	**0.01***
OFAV	4.57	32.04	0.48	9.12	**0.007***
	9.14	7.75	0.20	1.23	0.35
	13.72	9.85	0.23	2.43	0.15
	16.76	50.46	0.36	4.48	0.066
OFRA	4.57	10.75	0.32	3.69	0.066
	9.14	69.48	0.61	12.38	**0.011**
	13.72	72.11	0.82	36.80	**0.009**
	16.76	31.53	0.38	4.97	0.065
AAGA	4.57	14.07	0.33	3.98	**0.02**
	9.14	23.76	0.42	5.71	**0.03**
	13.72	16.22	0.12	1.09	0.31
	16.76	5.21	0.24	2.18	0.16
PAST	4.57	2.29	0.11	0.99	0.46
	9.14	0.03	0.00	0.00	1.00
	13.72	1.33	0.02	0.19	0.70
	16.76	0.80	0.02	0.15	0.81
PPOR	4.57	1.44	0.13	1.23	0.32
	9.14	5.09	0.29	3.32	0.11
	13.72	2.11	0.23	1.77	0.23
	16.76	3.66	0.19	1.69	0.25

Table S2. Generalized linear models for each species, comparing each metric against calculated irradiance at their collection depth. Significant values are indicated in bold with a star.

	M. cavernosa				*O. faveolata*				*O. franksi*			
**Metric**	**estimate**	**SE**	**z**	**p**	**estimate**	**SE**	**z**	**p**	**estimate**	**SE**	**z**	**p**
Corallite Density	2.79E-03	8.84E-04	3.15	**0.003***	1.02E-02	2.81E-03	3.62	**0.0009***	1.10E-02	2.59E-03	4.24	**0.0001***
Corallite Diameter	-5.30E-04	1.59E-04	-3.34	**0.002***	-1.77E-04	6.33E-05	-2.79	**0.008***	-2.55E-04	8.42E-05	-3.03	**0.004***
Corallite Area	-3.18E-04	1.04E-04	-3.07	**0.005***	-1.27E-04	3.90E-05	-3.27	**0.002***	-2.11E-04	5.84E-05	-3.61	**0.0008***
Calice Diameter	-5.06E-04	1.77E-04	-2.87	**0.007***	-7.79E-05	4.49E-05	-1.74	0.090	-8.86E-05	6.00E-05	-1.48	0.15
Calice Area	-5.93E-04	2.70E-04	-2.19	**0.036***	-3.50E-05	1.87E-05	-1.88	0.068	-4.35E-05	2.33E-05	-1.87	0.069
Host Protein	2.74E-04	4.42E-04	0.62	0.54	7.20E-04	5.72E-04	1.26	0.22	1.20E-03	5.62E-04	2.13	**0.040***
Host Biomass	2.29E-03	1.75E-03	1.31	0.20	3.55E-04	2.30E-03	0.15	0.88	9.69E-04	2.35E-03	0.41	0.68
Symbiont Biomass	8.51E-03	4.49E-03	1.90	0.066	4.43E-05	3.82E-03	0.01	0.99	4.45E-03	4.38E-03	1.02	0.32
Symbiont density	1.23E+03	3.72E+02	3.30	**0.002***	-8.13E+02	1.02E+03	-0.80	0.43	1.44E+03	1.08E+03	1.34	0.19
Chlorophyll a	2.50E-03	2.86E-03	0.87	0.39	-4.02E-03	3.98E-03	-1.01	0.32	3.55E-03	3.61E-03	0.98	0.33
Host $\delta^{13}C$	9.08E-03	1.92E-03	4.72	**0.0003***	1.49E-02	4.69E-03	3.19	**0.005***	4.87E-03	3.16E-03	1.54	0.14
Symbiont $\delta^{13}C$	-7.11E-04	4.94E-03	-0.14	0.89	1.44E-02	8.17E-03	1.77	0.094	-3.50E-03	1.11E-02	-0.31	0.76
Host $\delta^{15}N$	-7.48E-03	3.78E-03	-1.98	0.067	1.98E-03	1.63E-03	1.21	0.24	2.99E-04	1.58E-03	0.19	0.85
Symbiont $\delta^{15}N$	-2.83E-03	1.88E-03	-1.51	0.15	3.43E-03	1.96E-03	1.75	0.10	4.56E-04	2.21E-03	0.21	0.84
Host CN	1.48E-03	2.51E-03	0.59	0.57	-5.97E-03	3.06E-03	-1.95	0.068	-1.13E-03	3.84E-03	-0.29	0.77
Symbiont CN	-4.03E-03	5.00E-03	-0.81	0.43	-8.12E-03	9.19E-03	-0.88	0.39	-4.96E-03	1.08E-02	-0.46	0.65
	A. agaricites				*P. porites*				*P. astreoides*			
	**estimate**	**SE**	**z**	**p**	**estimate**	**SE**	**z**	**p**	**estimate**	**SE**	**z**	**p**
Corallite Density	1.65E-02	4.31E-03	3.84	**0.0004***	4.44E-02	9.18E-03	4.84	**0.00002***	4.73E-02	9.23E-03	5.12	**0.00001***
Corallite Diameter	-2.61E-04	8.50E-05	-3.07	**0.004***	-5.99E-05	2.67E-05	-2.24	**0.030***	-3.24E-05	2.48E-05	-1.31	0.20
Corallite Area	-1.46E-04	5.20E-05	-2.81	**0.008***	-1.89E-05	8.07E-06	-2.35	**0.024***	-7.08E-06	5.16E-06	-1.37	0.18
Calice Diameter	-7.63E-05	4.66E-05	-1.64	0.11	-1.13E-05	2.12E-05	-0.53	0.60	-9.70E-06	1.70E-05	-0.57	0.57
Calice Area	-1.40E-05	1.41E-05	-0.99	0.33	-2.03E-06	4.66E-06	-0.44	0.67	-1.85E-06	2.61E-06	-0.71	0.48
Host Protein	-2.22E-04	4.62E-04	-0.48	0.63	3.63E-03	1.45E-03	2.50	**0.017***	-3.34E-04	8.53E-04	-0.39	0.70
Host Biomass	-3.73E-03	1.70E-03	-2.19	**0.035***	5.71E-03	2.49E-03	2.29	**0.028***	7.63E-03	2.52E-03	3.03	**0.005***
Symbiont Biomass	1.01E-03	3.53E-03	0.29	0.78	1.06E-02	6.53E-03	1.63	0.11	-1.24E-02	5.97E-03	-2.08	**0.046***
Symbiont density	-2.22E+02	7.20E+02	-0.31	0.76	-2.29E+02	9.15E+02	-0.25	0.80	-7.75E+01	9.59E+02	-0.08	0.94
Chlorophyll a	-3.55E-03	3.22E-03	-1.10	0.28	1.74E-02	8.11E-03	2.15	**0.039***	-9.78E-03	5.38E-03	-1.82	0.078
Host $\delta^{13}C$	3.22E-03	4.26E-03	0.76	0.46	1.22E-02	2.49E-03	4.92	**0.0001***	1.43E-03	3.91E-03	0.37	0.72
Symbiont $\delta^{13}C$	4.33E-03	8.96E-03	0.48	0.64	1.06E-02	1.95E-03	5.42	**0.0001***	-1.43E-03	4.33E-03	-0.33	0.75
Host $\delta^{15}N$	-3.93E-03	2.64E-03	-1.49	0.16	1.99E-04	1.05E-03	0.19	0.85	-1.48E-03	1.73E-03	-0.86	0.40
Symbiont $\delta^{15}N$	3.83E-03	1.64E-03	2.34	**0.031***	1.15E-03	1.16E-03	0.99	0.34	2.26E-03	9.92E-04	2.28	**0.037***
Host CN	-3.67E-04	2.94E-03	-0.12	0.90	-3.67E-04	9.42E-04	-0.39	0.70	1.09E-04	2.61E-03	0.04	0.97
Symbiont CN	-7.33E-04	8.83E-03	-0.08	0.93	-9.55E-04	7.68E-04	-1.24	0.23	-6.59E-03	4.28E-03	-1.54	0.14

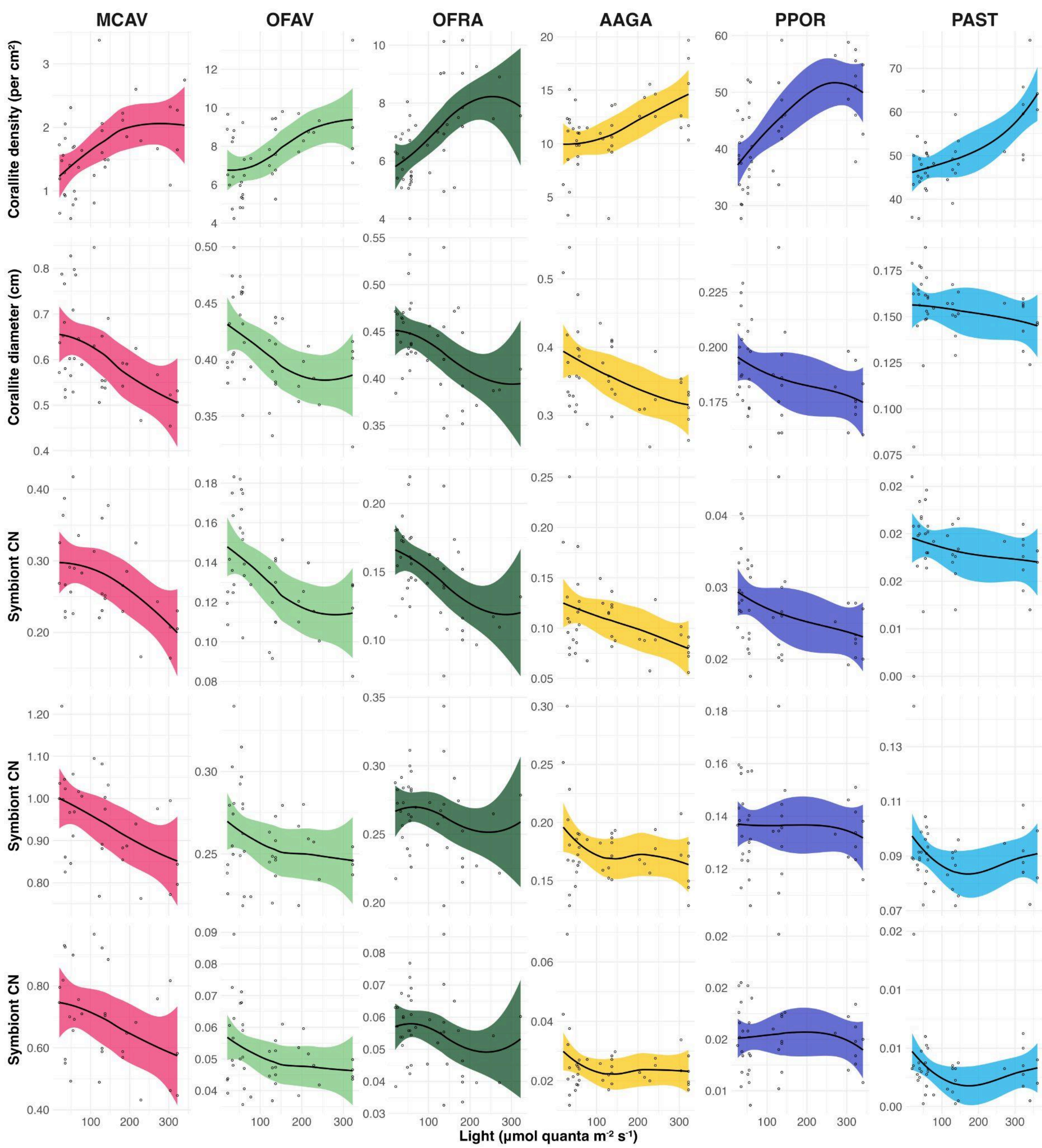

Figure S4. Skeletal morphology metrics for six species of Caribbean coral across a light gradient. The relationship between each metric and light availability was illustrated by creating a LOESS curve with a span of 1.2 and the bands of color represent the 95% confidence interval around the curve.

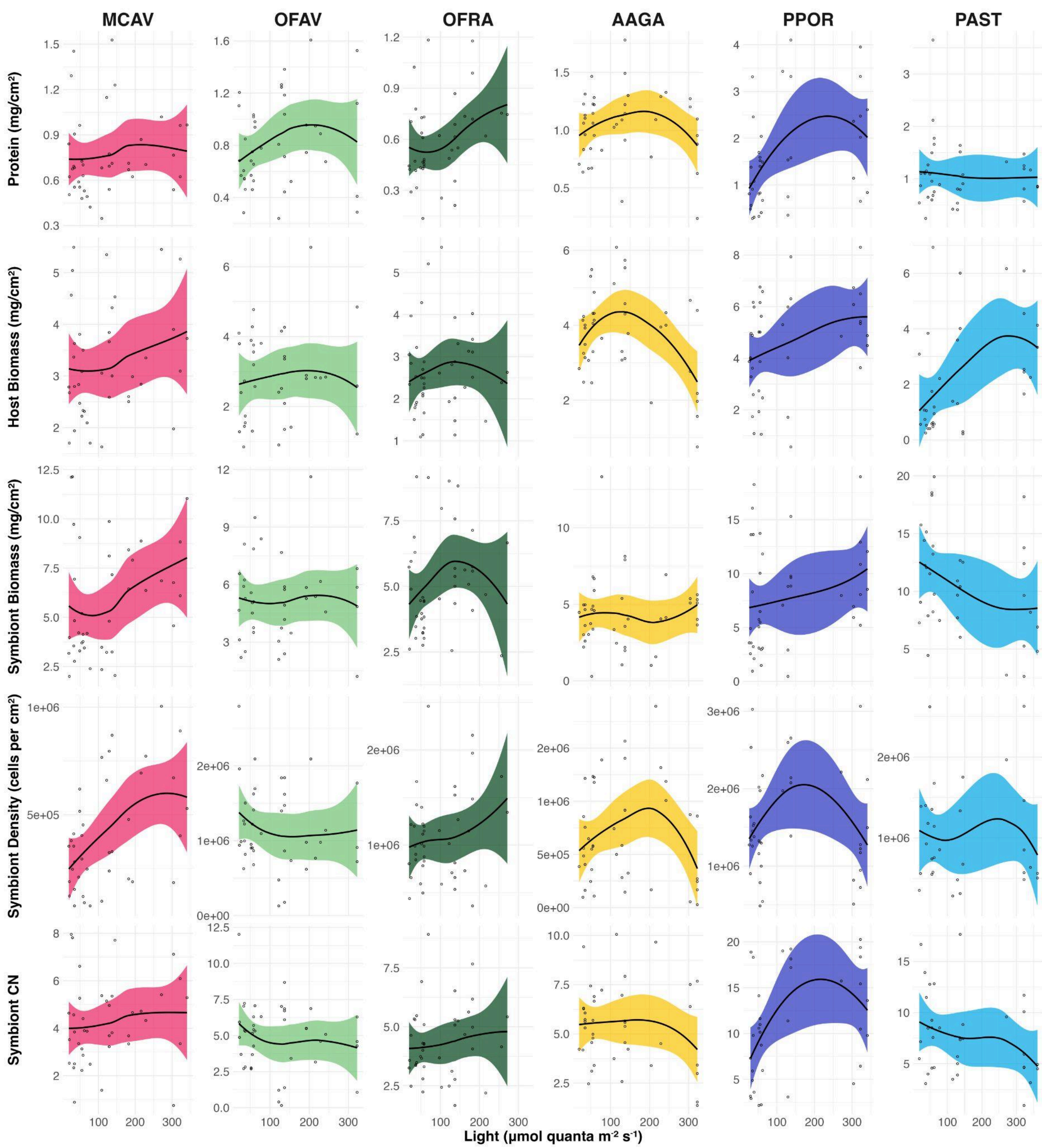

Figure S5. Physiology metrics for six species of coral across a depth gradient. The relationship between each metric and light availability was illustrated by creating a LOESS curve with a span of 1.2 and the bands of color represent the 95% confidence interval around the curve.

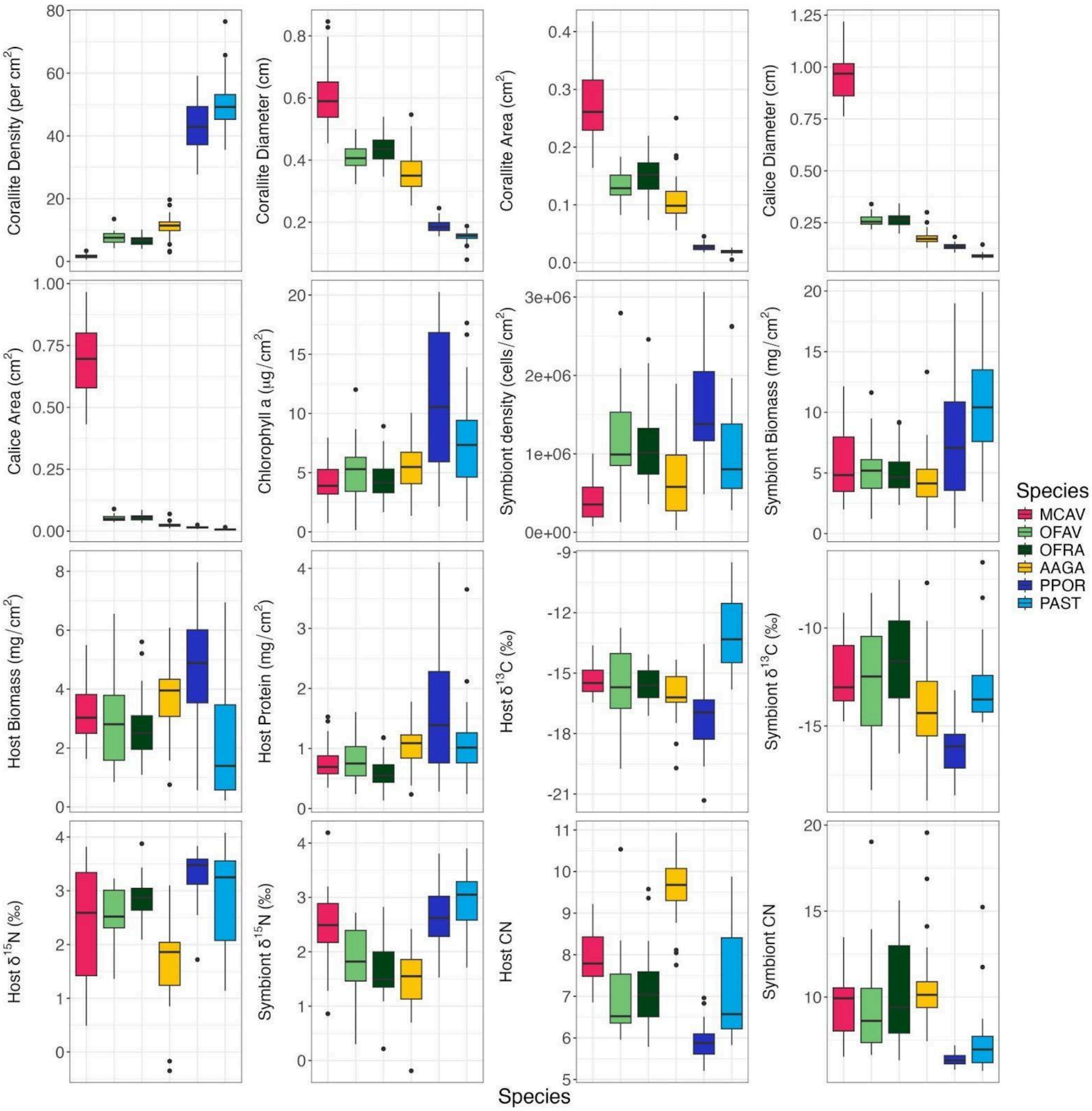

Figure S6. Raw values for each metric across all species with all depths pooled. Boxplot center lines display the median, and box edges represent the first and third quartile limits. The whiskers show the maximum and minimum values within 1.5 times the interquartile range and black dots represent outliers.

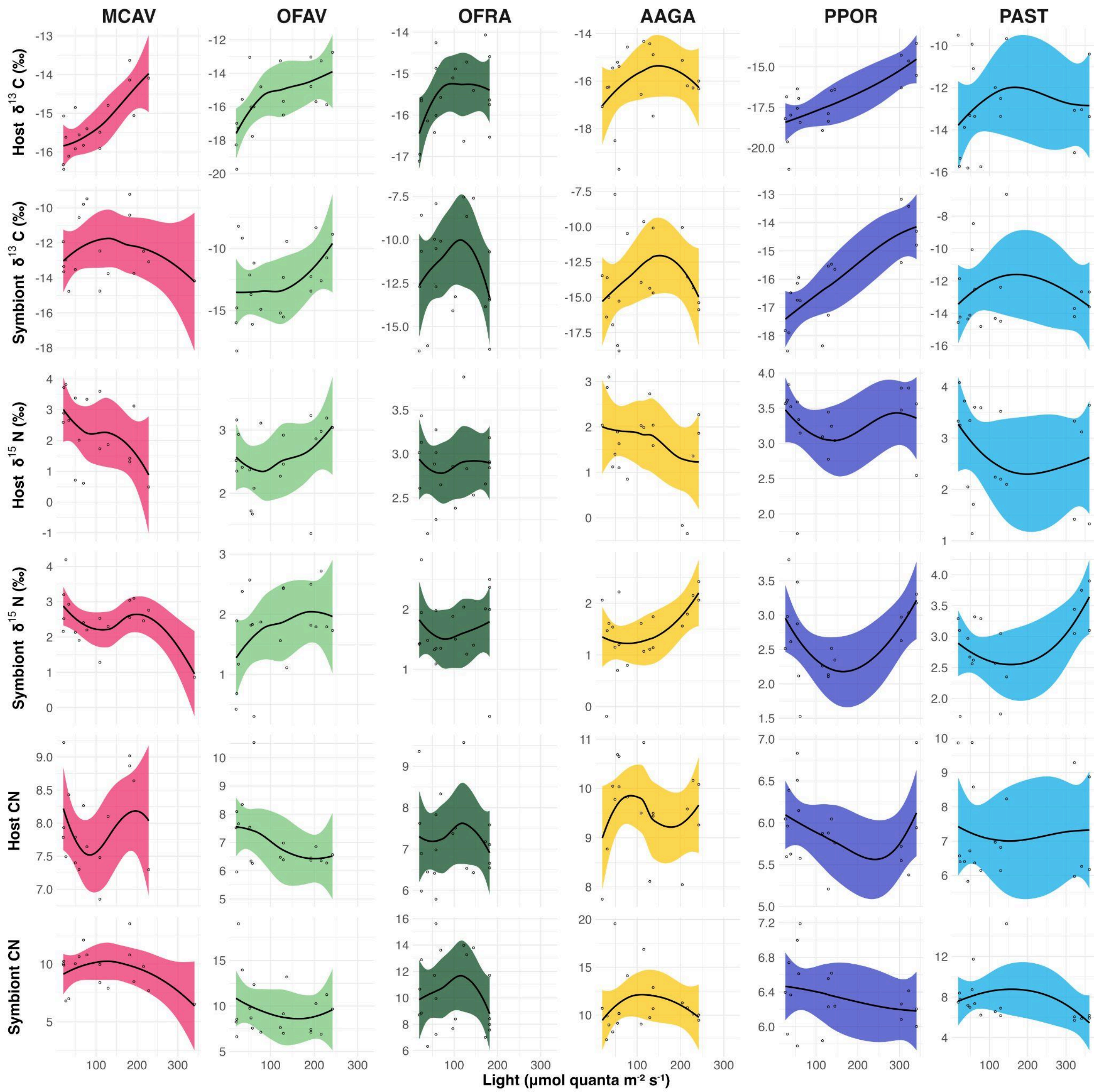

Figure S7. Bulk carbon and nitrogen isotopes for the host and symbionts of six species of Caribbean coral across a light gradient. The relationship between each metric and light availability was illustrated by creating a LOESS curve with a span of 1.2 and the bands of color represent the 95% confidence interval around the curve.

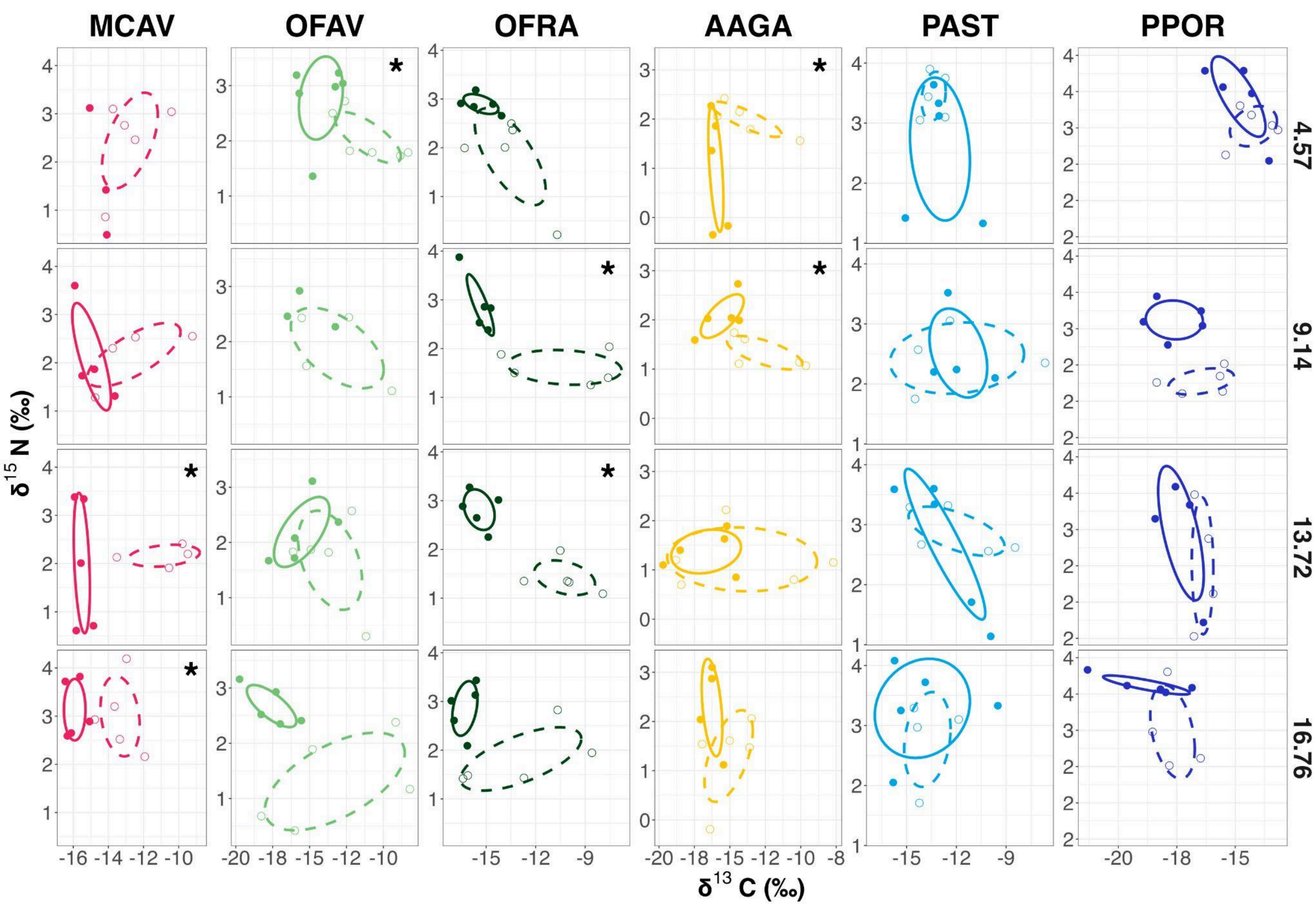

Figure S8. Isotope biplots of the relationship between the coral host (filled dots, solid line) and its symbionts (empty dots, dotted line) for six Caribbean species, separated by depths (indicated on the right axis). Black dotted lines illustrate the convex hulls of each group, indicating the total area of each isotopic niche. Ellipses represent the 40% confidence interval of each isotopic niche based on SIBER analysis. Ellipses are absent when fewer than four data points are available. Stars indicate host and symbiont pairs that occupy significantly different isotopic niches according to our PERMANOVAs (Table S1).

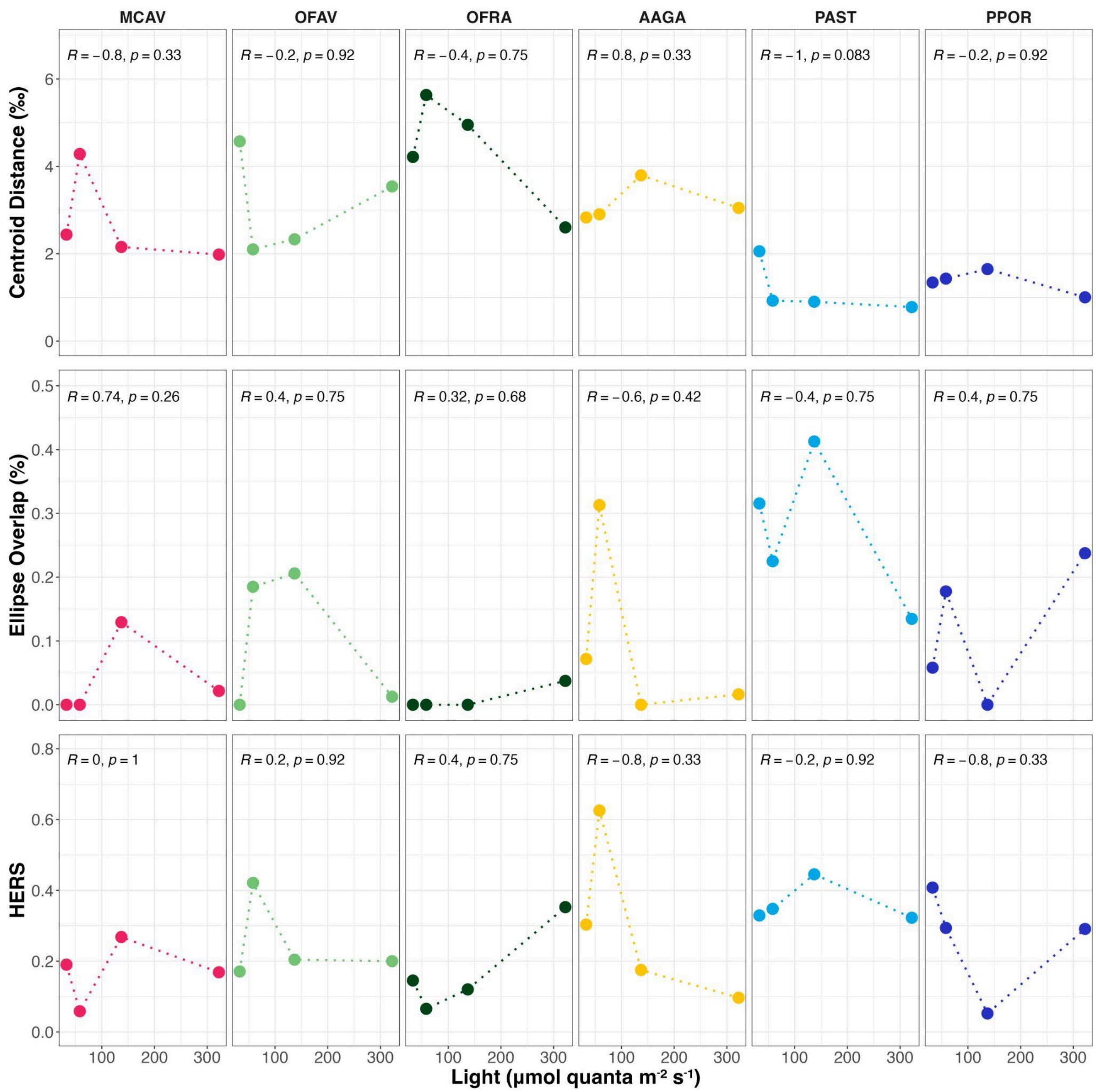

Figure S9. The centroid distance, ellipse overlap, and HERS scores taken from SIBER analyses did not change across the light gradient in any of the six species. This indicates that the trophic strategy of each species remains relatively constant across the light gradient. The summary statistics of each Spearman's rank correlation are displayed within each graph. Dots indicate values for each depth bin with dotted lines connecting them across the light gradient.
